# Deep learning-aided inter-species-comparison reveals shared and distinct molecular patterns in cynomolgus monkey and humans following non-specific T cell activation

**DOI:** 10.3389/fimmu.2025.1603716

**Published:** 2025-12-12

**Authors:** Vincent D. Friedrich, Kari Neier, Kristina Müller, Birgit Fogal, Zuzana Loncova, Michael Rade, Muhammad Shoaib, Ulrike Köhl, Kathleen Hoyt, Parimal Pande, Lily Blanchard, Ernest Raymond, Markus Scholz, Kristin Reiche, Holger Kirsten

**Affiliations:** 1 Institute for Medical Informatics, Statistics, and Epidemiology, University of Leipzig, Leipzig, Germany; 2 Center for Scalable Data Analytics and Artificial Intelligence (ScaDS.AI), Leipzig, Germany; 3 Boehringer Ingelheim Pharmaceuticals, Inc., Ridgefield CT, United States; 4 Biocenter, Institute of Bioinformatics, Medical University of Innsbruck, Innsbruck, Austria; 5 Fraunhofer Institute for Cell Therapy and Immunology, Leipzig, Germany; 6 Luxembourg Centre for Systems Biomedicine, University of Luxembourg, Belvaux, Luxembourg; 7 Cancer Center Central Germany (CCCG) Leipzig-Jena, University Hospital of Leipzig, Leipzig, Germany; 8 Institute for Clinical Immunology, Leipzig University, Leipzig, Germany; 9 Faculty of Mathematics and Computer Science, University of Leipzig, Leipzig, Germany

**Keywords:** ScRNA-seq, deep learning, cross-species analysis, translational gap, cynomolgus monkey, human, PBMC, immune response

## Abstract

The early phase of drug development relies on the examination of the efficacy and safety of therapeutic agents in animal models. Due to their close genetic and physiological relation to humans, cynomolgus monkeys (*Macaca fascicularis*) are a promising animal model in preclinical studies investigating the immune system. However, the shared and divergent characteristics of the immune response at the molecular level are not yet fully understood, which makes transferring findings from these studies to human conditions challenging. Here, we demonstrate a cross-species analysis pipeline using single-cell transcriptomics (scRNA-seq) data from peripheral blood mononuclear cells (PBMCs), investigating the transcriptomic response in cynomolgus monkeys and healthy humans following anti-*CD3*/anti-*CD28* T cell activation. For this, PBMCs were collected at baseline, stimulated *in vitro*, and measured at 0 hours, at 6 hours and at 24 hours post-stimulation, with two biological replicates per species. The analysis integrates Variational Autoencoder (VAE)-based deep learning, cell-cell communication, differential gene expression, and pathway enrichment for an in-depth data exploration. We observed shared molecular patterns across species in the transition from innate to adaptive immune response, such as the increase of CD4^+^ T cell proportion and the reduction of CD14^+^CD16^-^ and CD14^low^CD16^+^ monocytes. Specific transcriptional clusters related to metabolic reprogramming emerged in CD8^+^ T cells and related to inflammatory and antiviral programs in NK cells at 24 hours post-stimulation in both species, with stronger regulation of pathways related to cell cycle progression, DNA replication, and GPCR signaling in the emerging CD8^+^ T cell cluster in monkeys than in humans. Cross-species overlap in activated pathways increased from 6 to 24 hours post-stimulation, with pathway co-enrichment and shared foreground genes becoming more similar across species at 24 hours, including *Regulation Of Natural Killer Cell Chemotaxis* and *Interleukin-27-Mediated Signaling Pathway*. Across time, we observed a consistent decline in the expression of receptors and ligands involved in cell-cell communication in most cell types, however, the initial levels were higher in humans and the decline more pronounced. Our proposed computational framework enables systematic cross-species time series analyses, advancing translational research and contributing to improved development of immunomodulating therapies.

## 1 Introduction

Animal models are employed in preclinical research to study immune responses to specific stimuli or treatments (1, 2). However, the transfer of findings from animal studies to human application is constrained due to genetic, physiological, metabolic and immunological differences between species and due to the controlled conditions in laboratories (3). This translational gap has led to scenarios where promising results in animals could not be validated in humans (3). Even more, on some occasions, human responses to stimuli were not anticipated by preclinical animal models, including non-human primates (NHPs), and only became evident during human trials, i.e. at a late stage of drug development where considerable investments have already been made. For example, the anti-*CD28* monoclonal antibody *TGN1412*, designed to stimulate T cells *in vivo*, showed no proinflammatory effects in preclinical models including cynomolgus monkeys, but induced a severe inflammatory response in form of a cytokine storm in six healthy volunteers in the phase 1 clinical trial (4, 5). Similarly, in a clinical study aimed at treating chronic hepatitis B with fialuridine, the toxic reaction that led to the deaths of five out fifteen treated patients was not anticipated based on preclinical animal studies, again including cynomolgus monkeys (6, 7). Despite their close phylogenetic and physiological traits with humans, the infrequent use of NHPs - driven by ethical considerations - results in uncertainties regarding comparative immune responses (8). These knowledge gaps encompass interspecies incompatibilities and uncertainties about final transferable doses, creating significant translational challenges that ultimately hamper accurate prediction of severe side effects in clinical trials.

In our study, we explore similarities and differences of immune response in cynomolgus monkeys (*Macaca fascicularis*) and healthy humans (*Homo Sapiens*) following anti-*CD3*/anti-*CD28* T cell activation, a common method to trigger an immune response through selective T cell stimulation (9). Our goal is not only to gain biological insights but to establish a principled approach for mapping temporally resolved high-dimensional transcriptomic data between species. Since peripheral blood mononuclear cells (PBMCs) are composed of core immune cells, including lymphocytes and monocytes, they are considered a representative sample of the overall activity of the immune system (10). When analyzed with single-cell RNA sequencing (scRNA-seq), this enables a detailed assessment of immune activation and regulation at the cellular level, a method that has been employed in numerous studies (11–14). Moreover, the high resolution of scRNA-seq enables computational analysis at the gene level, the pathway level and across the transcriptome. For the latter, deep learning techniques such as variational autoencoders (VAEs) (15) are well suited, since they offer meaningful low-dimensional representations of high-dimensional gene expression, focussing on the most relevant features of the transcriptome. A VAE model consists of an encoder neural network and a decoder neural network, typically with the same architecture. The encoder maps the high-dimensional gene expression data to a compact low-dimensional latent representation, while the decoder takes the latent representation as input and reconstructs gene expression from it. This interaction between the encoder and decoder along with the underlying probability distribution in latent space encourages meaningful latent representations of the scRNA in trained VAE models, making the method widely used in scRNA data analyses (16, 17). Based on the *scGen* (18) VAE model implementation, we demonstrated in this study how VAE-generated representations can be used for *in-silico* humanization of transcriptomic data from cynomolgus monkeys and compared the transcriptomic dynamics at the cellular level over time across species. Additionally, we complemented our VAE-based analyses with examinations of cell signaling patterns, differential gene expression and pathways enrichment, thereby providing a comprehensive cross-species comparison of the gene programmes following non-specific T cell activation. Overall, our principled analysis strategy including deep learning provides a robust method for identifying both shared and diverging molecular patterns in cynomolgus monkeys and humans, thereby contributing to narrowing the translational gap between animal models and humans.

## 2 Results

### 2.1 Cross-species integration and cluster-based annotation

**Figure 1:**
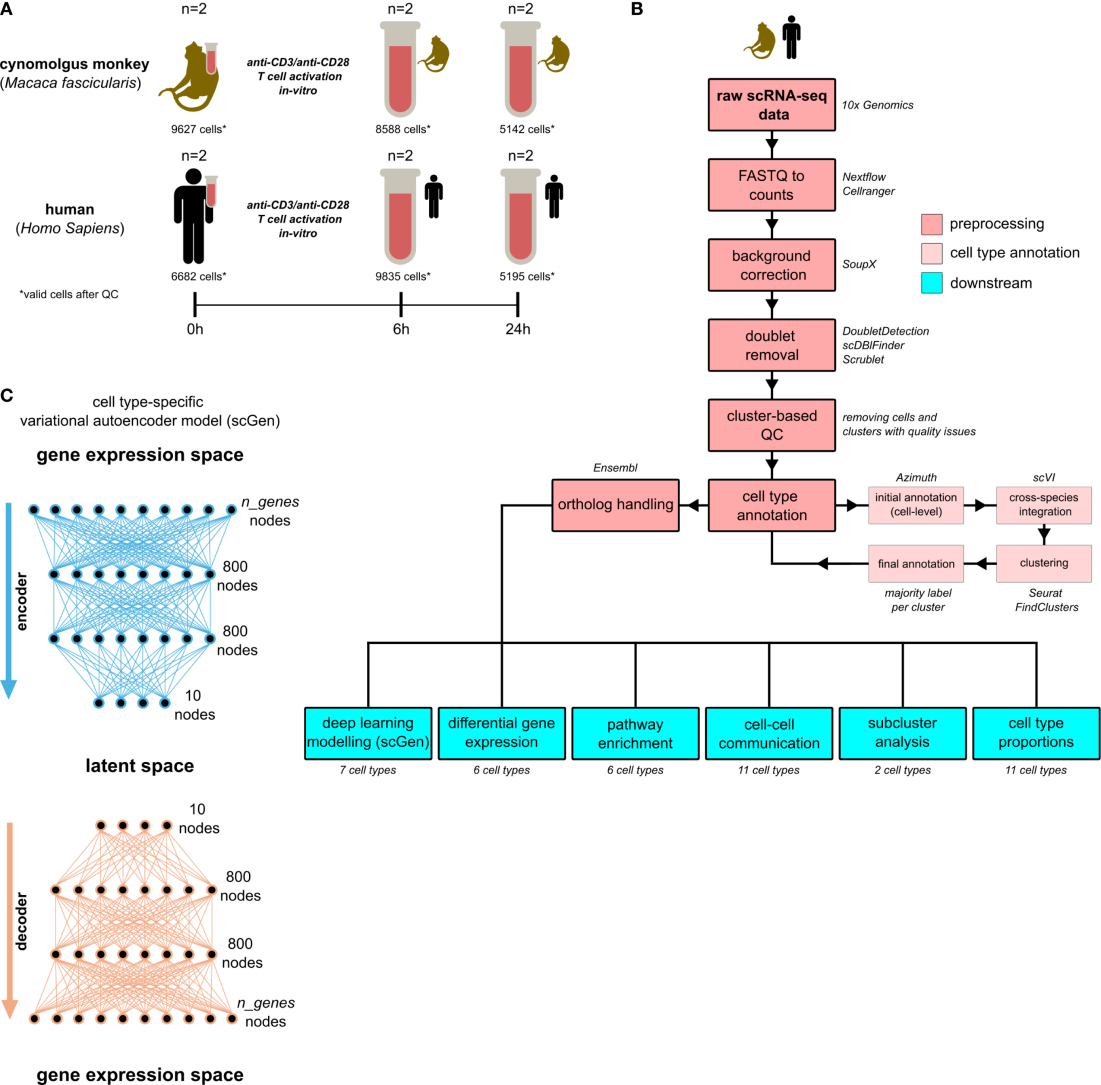
Overview. **(A)** Experimental design and sampling scheme. **(B)** Main analysis steps in preprocessing including cell type annotation and downstream analyses. **(C)** Schematic of the *scGen* variational autoencoder model architecture for each cell type used in deep learning modelling. QC, quality control; h, hours. Test Tube (“Reagenzglas”) icon by Delesign Graphics, licensed under CC BY 4.0. Original from iconscout.com.

We analyzed a total of 45,069 peripheral blood mononuclear cells (PBMCs) after quality control (QC) from two cynomolgus monkeys (*Macaca fascicularis*, 23,357 cells) and two humans (*Homo Sapiens*, 21,712 cells) using scRNA-seq at baseline and following anti-*CD3*/anti-*CD28* T Cell activation *in vitro*, with assessments performed at baseline (16,309 cells), after six hours (h) (18,423 cells) and after 24h (10,337 cells) (**Figure 1A**). Raw scRNA-seq data were processed to generate gene expression count matrices, followed by background correction, doublet removal, and cluster-based quality control, all performed at the species-level (**Figure 1B**, Material and methods 9.4, 9.5). In cynomolgus monkeys, QC resulted in the removal of 2,287 out of 25,644 cells (8.9%), of which 1,823 were identified as doublets. In humans, 2,179 out of 23,891 cells (9.1%) were removed, with 1,260 of those identified as doublets. Human orthologs were available for 81.3% of genes in our cynomolgus monkey data.

**Figure 2:**
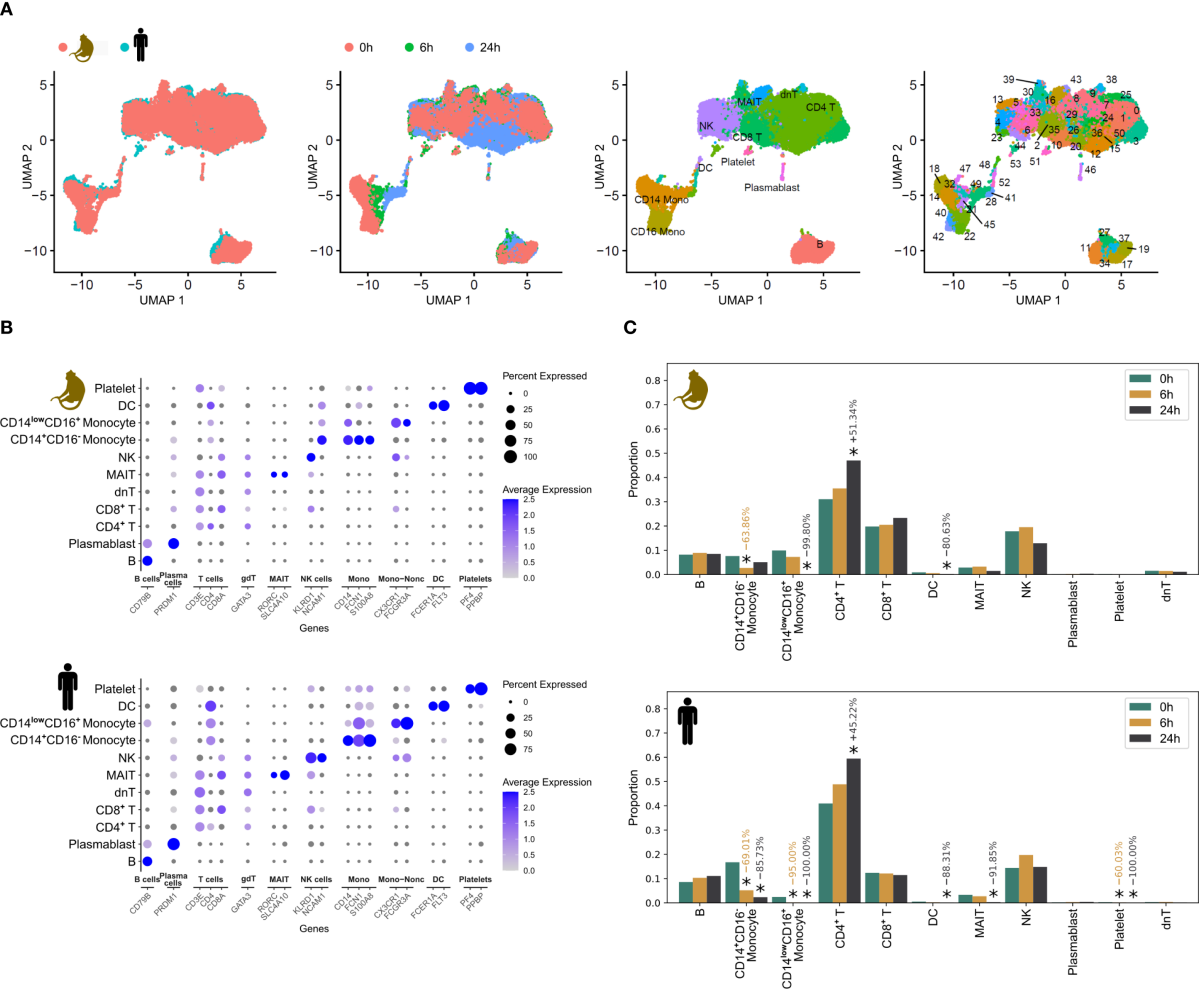
Integration of cross-species scRNA-seq data. **(A)** Uniform manifold approximation and projection (UMAP) plot of integrated cross-species scRNA-seq data with panels from left to right colored by species, timepoint post immune stimulation, cell type and clustering. Each cluster shown in panel 4 is assigned to the majority cell type label, shown in panel 3, based on the initial Azimuth-based automated annotation of individual cells within that cluster. **(B)** Gene expression profiles for selected cell type marker genes in cynomolgus monkey (upper panel) and human (lower panel) data. **(C)** Cell type proportions across temporal conditions in cynomolgus monkey (upper panel) and human (lower panel) data using data of all timepoints. A star indicates a statistically credible change in proportion, calculated using the *scCODA* framework in python. The number above the star shows the relative change in proportion compared to baseline (0h). h, hours.

After jointly embedding cynomolgus monkey and human data using *scVI* (19) with standard hyperparameters, we refined an initial automated cell type annotation from *Azimuth* (20) by assigning clusters derived from the *scVI* embedding to their majority cell type (**Figure 1B**, Material and methods 9.6), providing the foundation for downstream analyses (**Figures 1B, C**). In contrast to the unintegrated data (**Supplementary Figure S1**), the variance after integration was largest for cell types, followed by immune stimulation conditions, and then species (**Figure 2A**), indicating successful integration.

Next, we analyzed the expression of cell-type specific marker genes at the species-level to demonstrate the validity of the cluster-based cell type annotation. Overall, we observed similar expression patterns across key cell type markers in both species (**Figure 2B**).

While the initial proportion of lymphocytes differed between cynomolgus monkeys and humans, we observed highly similar changes in response to immune activation 6h and 24h post immune stimulation: CD4^+^ T cells significantly increased while CD14^+^CD16^-^ and CD14^low^CD16^+^ monocytes significantly decreased, with a stronger decrease observed in humans. There were no credible changes in the proportions of CD8^+^ T cells, B cells and NK cells (**Figure 2C**).

### 2.2 *ScGen* VAE modelling enables robust *in silico* humanization of cynomolgus monkey transcriptomics

**Figure 3:**
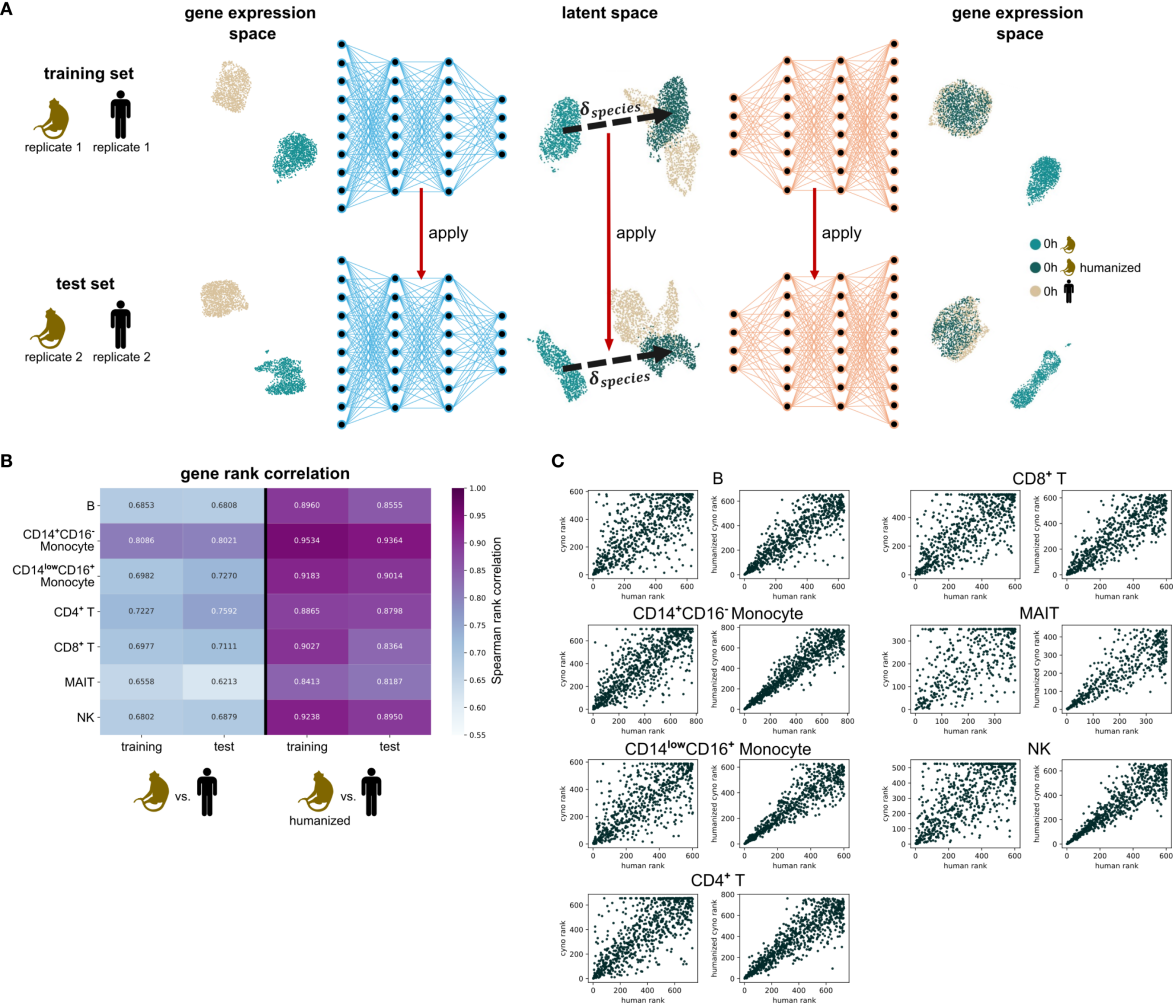
*In silico* humanization using VAE latent representation. **(A)** After splitting the cells at baseline (0h) into a training set and a test set, cell type-specific variational autoencoder (VAE) models are trained using the training set. Cell types with fewer than 1,000 cells are excluded from VAE modelling. Per cell type, the species-shift-vector δ_species_ is calculated, representing the general transition from cynomolgus monkeys to humans in VAE latent space. The VAE models and δ_species_ are then applied to the unseen test data without additional training or fine-tuning. The application of δ_species_ to cynomolgus monkey data is referred to as *in silico* humanization. **(B)** Spearman rank correlation of the mean gene expression of genes between cynomolgus monkeys and humans and between *in silico* humanized cynomolgus monkeys and humans across cell types, respectively, evaluated on the training set and the test set with an applied non-zero expression filter for human genes. **(C)** Scatter plots comparing mean gene expression ranks on the test set. Per cell type, the left panel compares human gene ranks (x-axis) with cynomolgus monkey gene ranks (y-axis) while the right panel compares human gene ranks (x-axis) with *in silico* humanized cynomolgus monkey gene ranks (y-axis). cyno: cynomolgus monkey. h, hours.

We tested how effectively we could transform (“humanize”) cynomolgus monkey gene expression data into human-like data using two VAE models, *scGen* (**Figure 1C**) and *LDVAE* (21) (**Supplementary Figure S2A**), and evaluated how generalizable their results were. To identify the most appropriate architecture for our use case, we performed VAE-specific proof-of-concept tests. For this, we split the cells assessed at baseline (0h) into training and test sets each containing the cells of distinct individuals. We trained cell type-specific VAE models with both architectures, including only those cell types with at least 1,000 cells in total, and calculated species-shift vectors (**δ_species_**) that capture the key differences between monkey and human gene expression patterns in the latent space. After applying these species-shift vectors to transform monkey data (**Figure 3A**), we evaluated how “human-like” the transformed data had become by comparing gene expression patterns between original human cells, original monkey cells, and transformed monkey cells. For the *scGen* model, the transformed monkey data consistently showed stronger correlation with human data compared to the original monkey data, and this held true for both training and test sets (**Figure 3B**). The similar performance on test data suggests our models captured genuine species differences rather than just memorizing the training data or describing individual-specific patterns, which is further supported by gene expression rank scatter plots (**Figure 3C**). In contrast, we observed a considerably weaker performance for the *LDVAE* model, evidenced by a lower Spearman gene rank correlation between *in silico* humanized monkey data and human data across all cell types, compared to *scGen* (**Supplementary Figure S2B**, **Supplementary Table S5**). Therefore, we used the *scGen* model for subsequent analyses.

### 2.3 VAE modelling suggests immune stimulation effects dominate individual variation at 24 hours post-activation

We next examined how well *scGen* VAE models could capture and predict transcriptomic changes following non-specific immune stimulation. When testing the models’ ability to predict gene expression at 24h post-stimulation, we found they could effectively generalize these changes across different individuals in both species for all cell types but MAIT (mucosal-associated invariant T) cells. This was reflected by the lowest Spearman rank correlation between predicted and observed gene expression for MAIT cells in both species, compared to other cell types. However, for the 6h timepoint, generalization across individuals was considerably lower using the same evaluation measure with Spearman gene rank correlations for predicted versus observed expression (6h vs. 6h predicted) below those of the corresponding 0h vs. 6h comparisons for most cell types (**Supplementary Figures S3A, C**). To understand whether this was either driven by the model performance or inter-individual variation exceeding the effect of the treatment at 6h, we compared results between splitting data by biological replicate (**Supplementary Figure S3A**) versus random assignment of cells to the training and the test set (**Supplementary Figure S3B**). We observed that random splits performed on average better by inherently accounting for individual variation, with a mean Spearman rank correlation between predicted and observed (6h vs. 6h predicted) gene expression across cell types and species of <0.835 for replicate-based splits and of >0.875 for random split. This suggests that the lower generalizability at 6h was mainly driven by inter-individual variation exceeding the effect of the treatment. Intersample differences in the VAE representation were comparable between species, suggesting no strong sex effect, as the human samples included both sexes while the cynomolgus monkey samples included only males (**Supplementary Figure S4**).

### 2.4 VAE and differential expression analysis demonstrate cell type-dependent conservation of immune responses

We then compared the transcriptomic responses to non-specific immune stimulation across species using the VAE data representation. To quantify the transcriptomic changes detected in our VAE, we used the VAE transition vectors, which capture both the overall magnitude of change and a direction of change. This direction is best interpreted when two VAE transition vectors are compared (e.g., changes in human vs. changes in monkey), where a cosine similarity of 1 indicates identical and a cosine similarity of 0 indicates completely opposite changes in the transcriptome in response to stimulation.

**Figure 4:**
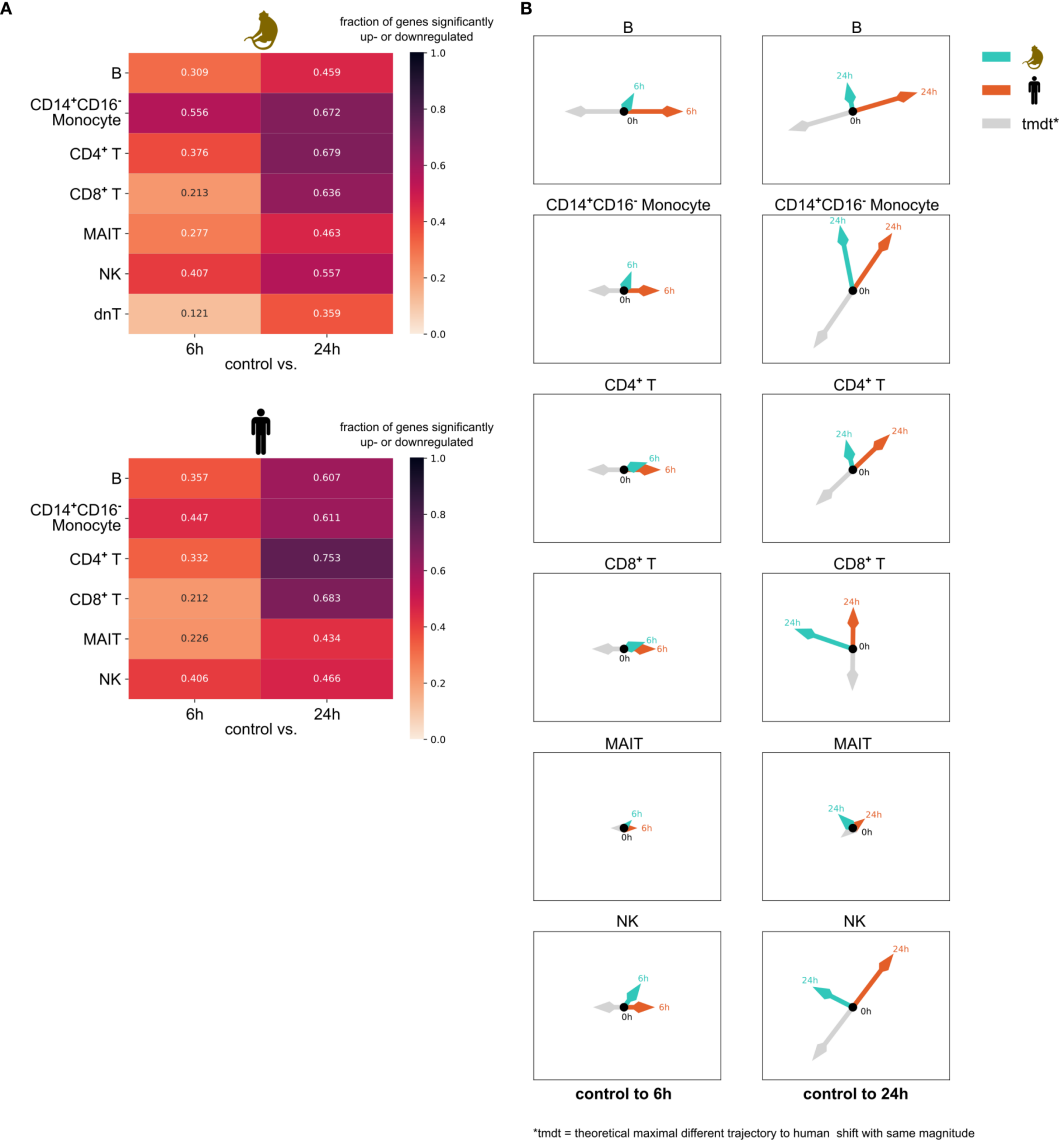
DGE and VAE perspective on transcriptome response following anti-*CD3*/anti-CD*28* T Cell activation. **(A)** Percentage of genes significantly up- or downregulated from differential gene expression (DGE) analysis in cynomolgus monkeys (upper panel) and humans (upper panel) at 6h and at 24h compared to baseline at 0h. **(B)** Two-dimensional representations of the 10-dimensional cell type- and species-specific temporal-shift-vectors in VAE latent space from control (0h) to 6h (left column) and 24h (right column), reflecting the trajectory of the transcriptome in the corresponding VAE latent space. The length of each arrow is proportional to its magnitude in the VAE representation and the angles between human and cynomolgus monkey vectors are derived from their pairwise cosine similarity. The angles between human vectors within a cell type reflect their pairwise cosine similarity. The grey vector represents the theoretical maximum difference in trajectory relative to the human shift vector, while maintaining the same magnitude. Tmdt: theoretical maximum difference in trajectory. h, hours.

By analyzing VAE transition vector’s magnitude and direction, we found stronger transcriptomic changes at 24h compared to 6h across all cell types (**Figure 4B**, **Supplementary Tables S11**, **12**). CD14^+^CD16^-^ monocytes showed similar responses between species at 24h (cosine similarity >0.8). In contrast, B cells and NK cells displayed notably different response patterns between species (cosine similarity <0.3), while CD4^+^ T cells maintained roughly similar response directions despite stronger regulation in humans (cosine similarity >0.7). Early responses at 6h showed high directional similarity in both CD4^+^ and CD8^+^ T cells (cosine similarity >0.9), though the magnitude of changes was small.

We compared these VAE-based results with the percentage of the transcriptome changed in response to stimulation calculated via the proportion of rejected null-hypotheses (22) in standard differential gene expression (DGE) analysis (**Figure 4A**), including cell types with at least five cells per timepoint across all replicates. These results were consistent with our VAE observations, as they also showed more extensive gene regulation at 24h than at 6h in all cell types. In particular, CD4^+^ T cells, CD8^+^ T cells and CD14^+^CD16^-^ monocytes showed substantial transcriptional changes, with >60% of genes significantly regulated at 24h in both species. The stronger regulation in human B cells compared to cynomolgus monkeys was also consistent with our VAE findings.

To further investigate the agreement between VAE-based and DGE-based analyses, we compared the importance of VAE features with the importance of these genes in DGE using Spearman rank correlation. This corroborated the agreement, as we found that all significant correlations (11 out of 11) between VAE feature importance and DGE rankings were positively correlated, and even among non-significant correlations, 11 out of 13 showed positive directionality, significantly more than expected by chance (p = 0.022, binomial test) (**Supplementary Figures S5A, B**). At the pathway level, this was similar, as key immune regulatory pathways identified in the VAE feature importance analysis, such as *Regulation Of Natural Killer Cell Chemotaxis*, were also enriched in the DGE analysis for most cell types (**Supplementary Figure S5C**).

### 2.5 Differential gene expression and pathway enrichment

**Figure 5:**
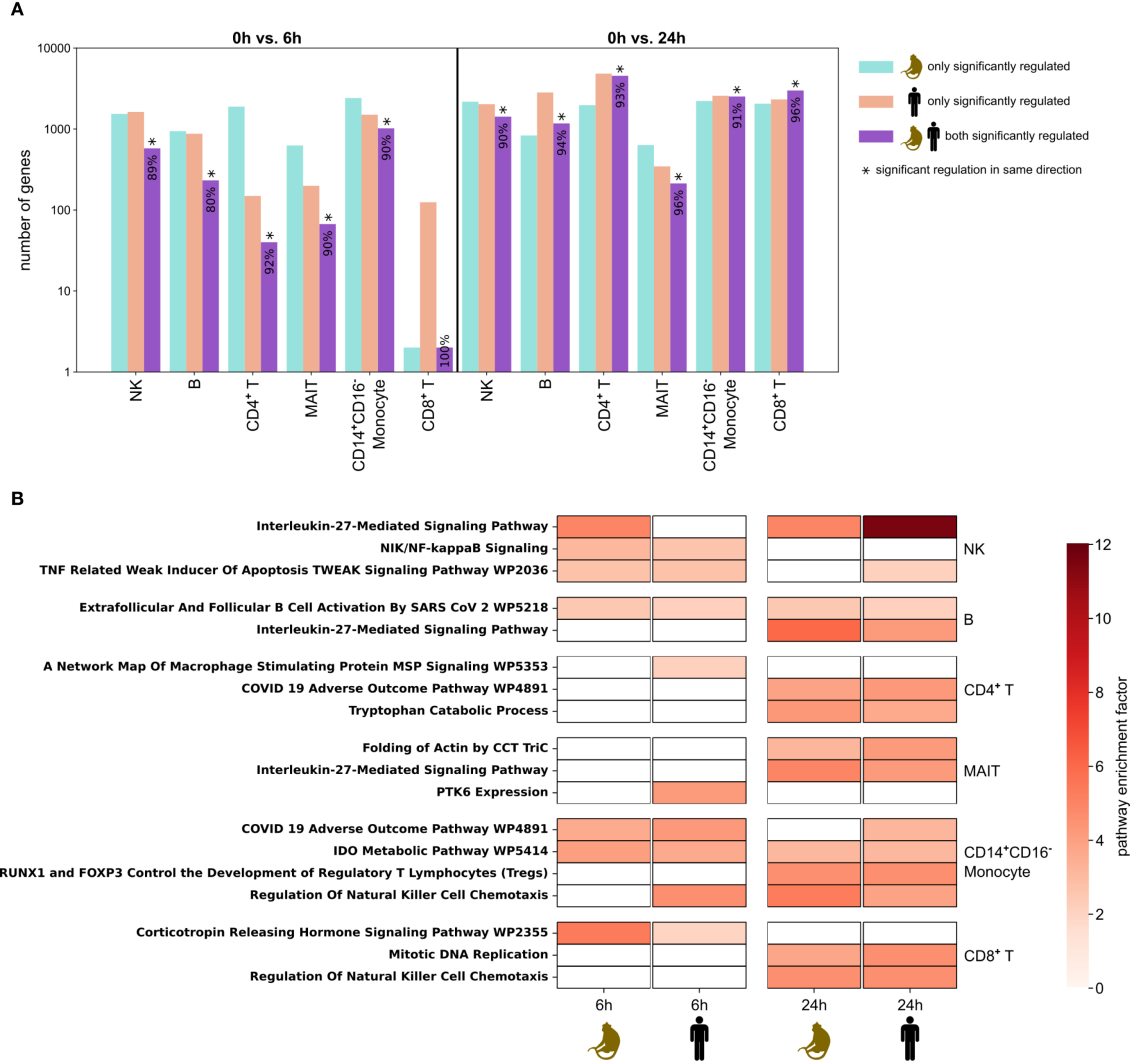
Differential gene expression and pathway enrichment analysis across species. **(A)** Barplot showing the logarithmic number of genes differentially expressed after 6h (left panel) and after 24h (right panel) in cynomolgus monkeys, humans and in both species at false discovery rate (FDR) ≤ 20%. In the bar for significant genes shared by both species, the percentage of genes regulated in the same direction is displayed. Statistical significance at p ≤ 0.01 for this co-regulation is indicated by a star and was calculated using prop.test function in R. **(B)** Top co-enriched pathways per celltype and condition at FDR ≤ 0.05, identified using the GSEApy Enrichr API with Reactome, WikiPathway, GO and KEGG databases. If no co-enriched pathways were present for a given cell type and condition, the top pathways within each species are shown. h, hours.

To better understand the co-regulatory mechanisms in cynomolgus monkeys and humans, we analyzed whether the same genes were differentially expressed across species (termed “co-regulated”) and, if so, whether the fold change was in the same direction (**Figure 5A**, **Supplementary Table S13**). Again, we observed a higher number of differentially regulated genes at 24h compared to 6h across all cell types at false discovery rate (FDR) ≤ 20%. When considering differentially genes not co-regulated across species, we observed a higher number of differentially regulated genes in cynomolgus monkeys at 6h and in humans at 24h. When considering differentially expressed genes at 24h, highest cross-species overlaps were observed in CD4^+^ T cells (4,549), CD8^+^ T cells (2,990), and CD14^+^CD16^-^ monocytes (2,526), with over 90% of these genes being regulated in the same direction. In CD14^+^ CD16^-^ monocytes, more than 1,000 genes differentially expressed at 6h were also overlapping, while in CD4^+^ and CD8^+^ T cells, this overlap was small. In CD4^+^ T cells, this was primarily due to the low number of differentially expressed genes in humans, suggesting an earlier response of CD4^+^ T cells in cynomolgus monkeys. To better understand differences and similarities in the temporal dynamics of DGE between cynomolgus monkeys and humans, we also compared asynchronous timepoints (0h vs. 6h in cynomolgus monkeys vs. 0h vs. 24h in humans, and vice versa). However, the overlap and agreement in fold-change direction of differentially expressed genes between species remained highest at the synchronous 24h post-stimulation timepoint (**Supplementary Figure S6**).

The top co-enriched pathways at FDR ≤ 5% across species were largely specific to immune response, cell signaling, and metabolic regulation, with a higher co-regulation at 24h (**Figure 5B**). In 59 out of 1,005 co-enriched pathways, the foreground gene sets were identical between species. For example, *CCL4*, *CCL3* and *XCL1* built the foreground in both species in the *Regulation Of Natural Killer Cell Chemotaxis* pathway enriched in CD8^+^ T cells at 24h, while *IFNG*, *IL2RA*, *TNFRSF18*, *CTLA4* and *NFATC2* formed the foreground in both species in the *RUNX1 and FOXP3 Control the Development of Regulatory T Lymphocytes (Tregs)* pathway enriched in CD14^+^CD16^-^ Monocytes at 24h. In contrast, only 4 out of 1,005 co-enriched pathways had completely different foreground gene sets with no genes shared between species. Overall, both the number of co-enriched pathways and the overlap of foreground genes were higher at 24h than at 6h (**Supplementary Figure S7****).**

### 2.6 Cell-cell communication

**Figure 6:**
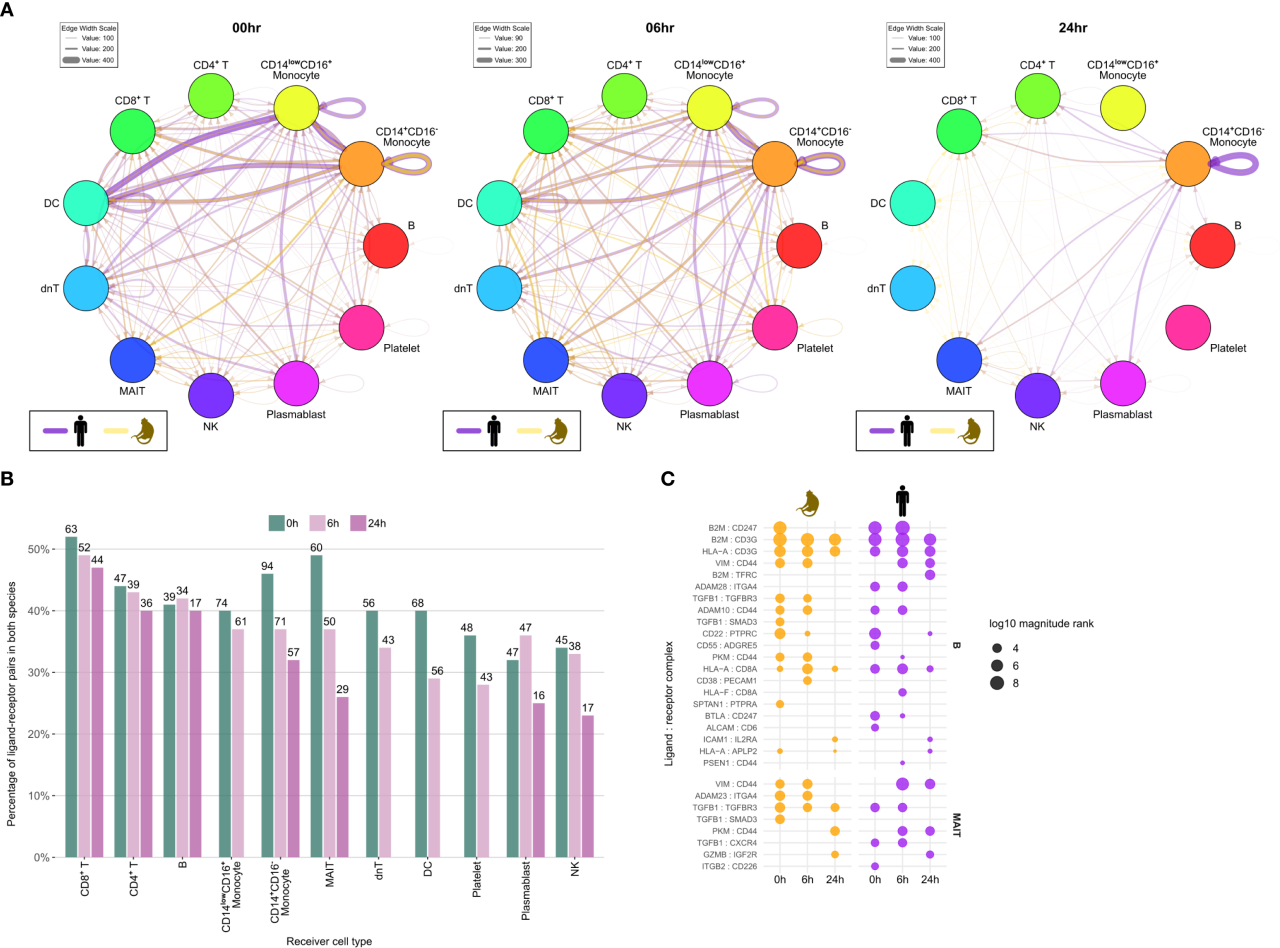
Cell-cell communication analysis reveals conserved signaling dynamics with temporal decline across species. **(A)** Network visualization of cell-cell communication at 0h, 6h, and 24h post-stimulation. Nodes represent cell types and directed edges represent significant ligand-receptor interactions (magnitude rank and specificity rank ≤ 0.001). Edge thickness indicates interaction strength (sum of log10 magnitude ranks). Purple edges indicate human interactions, gold edges indicate cynomolgus monkey interactions. **(B)** Percentage of significantly active ligand-receptor pairs showing cross-species conservation. Bars show the proportion of interactions that are shared between both species. Numbers above bars indicate the number of unique shared interactions. This overlap was always higher than expected by chance (Fisher’s exact test p<0.001) **(C)** Dot plot showing specific ligand-receptor pairs targeting CD8+ T cells where ligands were differentially expressed at FDR≤ 5% in the corresponding source cell types or receptors in CD8+ T as target cell type in any of the two species. Point size indicates interaction magnitude rank, and interactions are grouped by source cell type and timepoint. h, hours.

We analyzed cell-cell communication (CCC) using *LIANA* (23) to assess cell-type-specific signaling at baseline and over time following immune stimulation, comparing source vs. target activity and shared vs. species-specific responses. Here, source refers to cells emitting ligands and targets to cells emitting receptors. In general, the analysis of cell-to-cell communication revealed dynamic temporal patterns with considerable cross-species conservation. Network visualization showed that signaling activity was strongest at baseline (0h) across most cell types, with a consistent decline at six and 24 hours post-stimulation in both species (**Figure 6A**). Cell types related to innate immunity, such as monocytes and dendritic cells, exhibited the highest baseline signaling activity. This illustrates their dominant role in steady-state immune surveillance. This pattern was conserved across species, with humans showing particularly strong baseline communication intensity. Analysis of ligand-receptor pair conservation revealed substantial overlap across species, which was higher than expected by chance for all cell types and timepoints (p < 0.001; **Figure 6B**). The highest conservation was observed in CD8+ T cells (approximately 50% of all receptor-ligand pairs). However, the proportion of conserved interactions decreased to a certain extent for most cell types from 0h to 6h to 24h, suggesting that the resting state may be more conserved than the activated state following stimulation. A detailed analysis of CD8^+^ T cell-targeting interactions, filtered for cases in which either the ligands, the receptors, or both were differentially expressed, revealed similar patterns for classical interactions, such as *HLA-A:CD8A*, which is relevant for antigen recognition, and the *ADAM10:CD44* interaction, which is relevant for receptor shedding. However, different patterns were also observed, such as those seen in the *VIM: CD44* interaction, which is relevant for homing/retention and was more pronounced at later time points in humans (**Figure 6C**). When summing across receptors and ligands, conservations of signal dynamics across species, was confirmed, with source activity stronger than target activity and activity in humans stronger than in cynomolgus monkeys, especially at baseline and for monocytes, with humans showing particularly strong source activity (**Supplementary Figure S8**).

### 2.7 Cell type-specific cluster analysis

**Figure 7:**
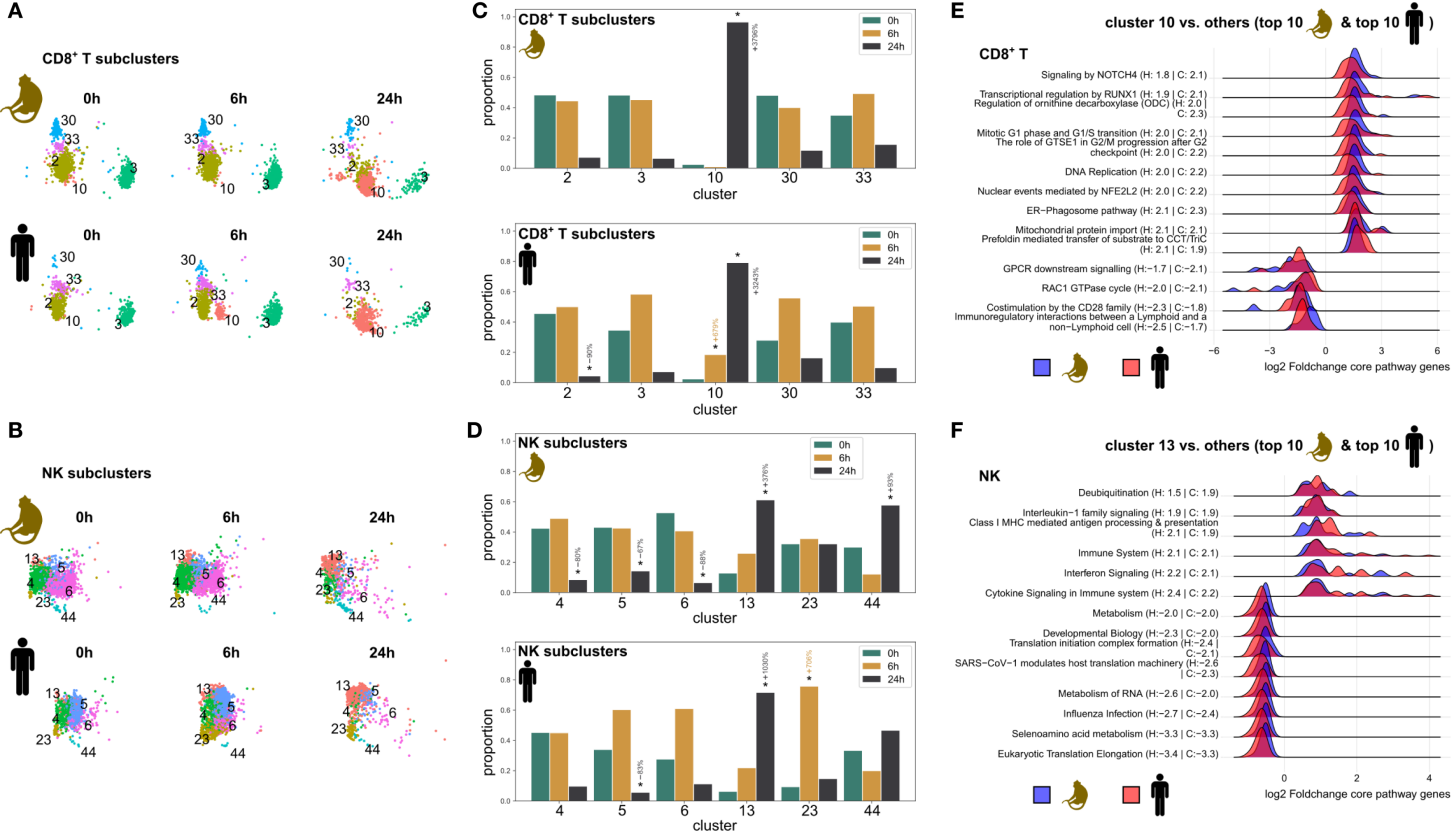
Cell subtype analysis. **(A, B)** UMAP representation of CD8^+^ T cell subclusters **(A)**, and NK cell subclusters **(B)** organized by timepoint and species. **(C, D)** Proportions of CD8^+^ T cell subclusters **(C)** and NK cell subclusters **(D)** across timepoints. A star denotes a statistically credible change in proportion, determined using the scCODA framework in python. The relative change in proportion compared to the baseline (0h) is highlighted for clusters and timepoints with credible changes. **(E, F)** Top 10 enriched pathways in cynomolgus monkeys and humans calculated for cluster 10 vs. all other clusters in the CD8^+^ T cell population **(E)**, and for cluster 13 vs. all other clusters in the NK cell population **(F)** h, hours; H, human; C, cynomolgus monkey.

To better understand the heterogeneity within specific immune cell populations, we analyzed how transcriptome clusters within cell types changed at 6h and 24h post stimulation for top abundant cell types without credible changes in the overall proportion. This pattern was most evident for CD8^+^ and NK cells (**Figure 7**). Specifically, in CD8^+^ T cells, we identified cluster 10, which became prominent at 24h post-stimulation in both species, increasing from nearly undetectable levels at baseline to become the dominant population (**Figures 7A, C**). This was even more pronounced in cynomolgus monkeys than in humans. Notably, this expansion occurred alongside a concomitant decrease in all other clusters, suggesting a general shift of CD8^+^ T cell states during non-specific activation. Pathway analysis of cluster 10 showed strong enrichment of pathways related to cellular processes including protein degradation (ER-phagosome), cell cycle regulation (G2/M progression, DNA Replication), and protein folding/import (*CCT/TriC*, mitochondrial protein import), suggesting high metabolic activity with increased protein turnover. Differential expression within these pathways was stronger in monkeys. In contrast, CD8^+^ T cells other than cluster 10 were enriched for immune pathways including *GPCR* signaling, *CD28* costimulation, immunoregulatory interactions, and *RAC1* GTPase cycle, characteristic for standard T cell activation programs (**Figure 7E**, **Supplementary Tables S6**, **8**).

NK cells showed a similar temporal reorganization, with cluster 13 emerging as the dominant population at 24h in both species (**Figures 7B, D**). The timing of this emergence was consistent across species, although a stronger expansion of cluster 13 appeared in humans compared to cynomolgus monkeys. Emerging NK cell cluster 13 showed strong enrichment of pathways related to immune activation and antiviral responses, including cytokine signaling, interferon signaling, and class I MHC-mediated antigen processing, with particularly increased expression of genes in the interferon and cytokine signaling pathway with genes such as *CCL3*, *CCL4*, *STAT1*, and *ISG15*, suggesting an activated inflammatory state. The other, shrinking clusters, showed enrichment of pathways related to more basic cellular functions, with increased expression of ribosomal and translational pathways (eukaryotic translation elongation, selenoamino acid metabolism), with lower differential expression in humans, suggesting that these cells remained in a more homeostatic state (**Figure 7F**, **Supplementary Tables S7**, **9**).

## 3 Discussion

This study presents a comprehensive cross-species analysis of immune activation in cynomolgus monkeys and humans using single-cell transcriptomics. By leveraging deep learning methods, cell-cell communication (CCC) analysis, differential gene expression (DGE), and pathway enrichment, we identified both shared and species-specific transcriptomic responses to anti-*CD3*/anti-*CD28* T cell stimulation, one of the key steps in immune-related adverse events (9). Our findings contribute to a deeper understanding of the molecular mechanisms underlying immune activation and highlight key differences that must be considered when translating preclinical findings to human contexts.

Our study integrates standard bioinformatics with AI representation learning. While standard bioinformatics emphasizes interpretability, AI models can capture complex patterns in an agnostic manner. In our case, we eventually applied the *scGen* variational autoencoder (VAE) model (18), a type of AI model that generates meaningful low-dimensional representations of the transcriptome; however, this approach lacks direct interpretability of the latent feature space. Some VAE architectures prioritize interpretability over latent space capacity (24). For example, the *LDVAE* model (21) employs a linear decoder to directly link the latent space to gene expression. Other VAE models, such as *OntoVAE* or *expiMap*, allow for the restriction of latent features to reflect the activity of specific biological processes (25, 26). Since the performance of different VAE architecture can considerably differ for a specific use case, which we also observed, we strongly recommend thoroughly validating the performance of the autoencoder specific for each dataset as we successfully did with the proof-of-concept approach.

In both, standard and AI-based methodology, ortholog handling is a crucial step in cross-species comparisons, as the common gene space forms the base for most downstream analyses (27). In our study, orthologs were available for more than 80% of all genes. This level of mapping might differ in genealogically more distant species, potentially affecting the performance of our proposed pipeline. To keep the information loss in ortholog handling as small as possible, we enabled our VAE approach to address the ambiguity of n-to-m gene assignments directly, by including all orthologs assigned to the same gene. We believe that continued progress in the field of genome annotation is expected to improve gene mapping across species, thus enhancing cross-species analyses of the transcriptome (28, 29).

When analyzing cell type proportions over time following non-specific immune activation (**Figure 2C**), we observed the expected dynamics in mononuclear cells (30). Specifically, while NK and CD8^+^ T cells maintained stable overall proportions, deeper analysis revealed a consistent cell stage transition at 24h post-stimulation: CD8^+^ T cells developed a metabolically reprogrammed state, while NK cells adopted an inflammatory and antiviral program. This pattern was observed across species, although quantitative differences in cluster responses exist such as a stronger metabolic programming in monkey CD8^+^ T cells.

DGE, CCC and VAE analyses revealed changes in the transcriptome also across other cell types, demonstrating that non-specific T cell activation affects multiple cell types while shifting the immune response from an innate-like to a more adaptive-like state, with similarities but also differences across species. Exemplary, CCC analysis suggested the hypothesis of a potential decline of cell signaling following immune stimulation, aligning with early T cell activation biology, where T cell stimulation triggers rapid T cell receptor (TCR) signaling, leading to cells shifting their transcriptional programs from maintenance to activation. Notably, humans exhibited higher baseline communication intensities (**Figure 6**). DGE and pathway enrichment indicated an increase in gene and pathway co-enrichment from 6h to 24h post-stimulation (**Figure 5**). Although we observed some evidence for a different temporal dynamic between cynomolgus monkeys and humans, similarity was highest 24h post stimulation across species, suggesting that the transcriptional trajectories might converge, at least partly, over time. The VAE-based analysis revealed greater transcriptomic divergences in B cells and NK cells over time compared to the more similar trajectories between cynomolgus monkeys and humans observed in CD4^+^ T cells and CD14^+^CD16^-^ monocytes (**Figure 4**). Thereby, findings of standard bioinformatic approaches and AI-driven analyses were consistent. For many cell types and conditions, we could further demonstrate a significant correlation between feature importance of genes used in the VAE and their statistical significance in DGE, even though the VAE captures in addition to immune-stimuli-related differences also species-specific differences (**Supplementary Figure S5**).

While our approach identified similarities and differences between a measured response to the same stimulus at the transcriptome level across species, it primarily serves as a first step introducing methodology and demonstrating their feasibility for such an interspecies comparison, as we also introduced generalizable measures how to quantify their performance (e.g. our proof-of-concept analysis shown in **Figure 3**). Looking ahead, further work with larger datasets, systematic validation, and expanded interpretability approaches should improve this strategy to provide robust molecular insights and even leverage the generative capabilities of VAEs to predict human-like transcriptional responses for conditions observed only in the animal data. For example, the model could be used to estimate human responses to stimuli or treatment levels not directly measured in humans but present in the animal data. Furthermore, the VAE’s interpretable latent space could enable interpolation between known conditions, for instance predicting a human transcriptomic response to an intermediate stimulus dose, based on training data from low and high doses.

This could support the design of more targeted, effective, and safer therapeutic interventions in humans, for example by helping identify dose ranges that reduce the risk of overstimulation-related adverse effects.

Comparing our study with Lin et al. (31), which examined phytohemagglutinin (*PHA*) and lipopolysaccharide (*LPS*) stimulation of PBMCs in cynomolgus monkeys and humans, we found that both our anti-*CD3*/anti-*CD28* T cell stimulation and *PHA* induced lymphocyte proliferation. However, *PHA* triggered stronger proliferation and broader cytokine secretion in humans compared to cynomolgus monkeys, especially for cytokine levels. Although *LPS* stimulation primarily targets innate immune response, anti-*CD3*/anti-*CD28* led also to increased CD4^+^ T cell proportions and decreased monocyte levels. Across all three immune stimulation methods, enriched differentially expressed genes were linked to common biological functions, including immune response and chemotaxis. While *PHA* induced significantly stronger proliferation in humans than in monkeys with about nine times more differentially expressed genes, this effect was not observed with *LPS* or anti-*CD3*/anti-*CD28*, where differences were more subtle. Notably, our study utilized scRNA-seq with measurements at 6 and 24 hours, enabling detailed cell type-specific temporal analysis, whereas Lin et al. employed bulk RNA-seq with a single assessment at 72 hours focusing on overall PBMC response.

### 3.1 Limitations

While our study provides valuable insights into cross-species non-specific immune responses, several limitations must be acknowledged. As a proof-of-concept investigation, we prioritized temporal resolution by including three time points (0h, 6h, and 24h post-stimulation) over biological triplicates, allowing us to capture better dynamic cell activation while minimizing experimental burden on animal subjects. However, future studies with larger cohorts will be essential to validate these findings. Still, this design allowed to compare the inter-individual variation with the immune treatment effect, while processing of all samples in a homogeneous lab environment contributed to minimal batch effects observed (**Supplementary Figure S1**). Second, our analysis focused on mRNA expression, excluding non-coding RNAs, microRNAs, and other omics layers such as proteomics or metabolomics, which could provide deeper insights into immune regulation and might be integrated in future analysis of immune dynamics across species. Furthermore, the 10x v3Genomics platform used in our study captures only about a third and not the entirety of the transcriptome. Third, our study uses *in vitro* non-specific stimulation lacking the full physiological complexity of *in vivo* models. However, this approach enables better experimental control, addresses a key step in adverse- and ex-vivo immune stimulation and reduces animal and human burden while still providing valuable mechanistic insights. Fourth, not all cell types could be included in every downstream analysis due to differing methodological requirements, for instance for the VAE and for DGE.

### 3.2 Conclusions

Our cross-species analysis of immune activation in cynomolgus monkeys and humans using scRNA-seq revealed both shared and species-specific transcriptomic responses. Key similarities included increased CD4^+^ T cell and decreased monocyte proportions, along with higher shared gene and pathway enrichment across species 24h post-activation. Transcriptomic trajectories analyzed through VAE in B cells and NK cells showed greater divergence than in other cell types with changes 24h post-stimulation surpassing inter-individual variations. Cell-cell communication declined post-stimulation, with humans exhibiting higher baseline levels. The principled analysis demonstrated the synergistic potential of standard bioinformatics and AI-based methods of representation learning, enhancing translational research and advancing the development of immunomodulating therapies.

## 4 Materials and methods

### 4.1 PBMC isolation

Peripheral blood mononuclear cells (PBMCs) were isolated from anonymized healthy adult blood (N = 2; 1 male, 1 female) collected by Sanguine at Boehringer Ingelheim in Ridgefield, CT, USA. PBMCs were also isolated from cynomolgus monkey (*Macaca fascicularis)* blood (N = 2; 2 males), originating from Bioculture Mauritius c/o Bioculture US LLC, Immokalee, FL. Sex was inferred from expression levels of (non-PAR) Y-chromosome markers (*DDX3Y*, *KDM5D*, *USP9Y*, *UTY*, *ZFY*, *EIF1AY*, *NLGN4Y*, and *RPS4Y1*). Cynomolgus monkeys were housed at an AAALAC accredited institution, Boehringer Ingelheim in Ridgefield, CT, USA, with approval of the Institutional Animal Care and Use Committee (IACUC).

In detail, 6ml blood with equal volume of 1x PBS + 2% FBS was diluted from each donor. A 90% lymphoprep solution was prepared with PBS and 15ml lymphoprep solution was added to each SepMate-50 tube. After centrifugation at 1200xg for 5 minutes, diluted blood was layered 1:1 with 1xPBS +2% FBS into each tube. The tubes were centrifuged at 1200xg for 10 minutes. PBMC and plasma from each donor was transferred to a new 50ml tube using pipette and centrifuged at 300xg for 8min. Next, the fraction including PBMCs was resuspended in 10ml ACK lysis buffer and incubated at room temperature for 5 minutes. 20ml 1xPBS +2% FBS were added, following centrifugation at 300xg for 8 minutes. PBMCs were resuspended in 10ml ACK lysis buffer and incubated at room temperature for 5 minutes. 20ml 1xPBS +2% FBS were added and tubes were centrifuged at 300xg for 8 minutes, and the cells were resuspended in 10ml RPMI1640 working Media. The Guava Count machine was used for cell counting. It was cleaned, quality checked and run for each sample. The samples were centrifuged at 300xg for 8 minutes. The cells were resuspended in RPMI1640 working media to a concentration of 200,000 cells/ul (cynomolgus monkey) and 250,000 cells/ul (human).

### 4.2 Antibody stimulation

To ensure similar level of stimulation in both species, cells were stimulated with anti-*CD3* antibodies at concentrations of 10 ng/mL for humans and 1000 ng/mL for cynomolgus monkeys and with anti-*CD28* antibodies at a concentration of 95 ng/mL for both species. Cells were stimulated for 6 or 24 hours in 12-well and 96-well tissue culture plates in cynomolgus monkeys and humans, respectively. The used reagents are outlined in **Supplementary Note 1**.

### 4.3 Generation of single-cell sequencing libraries

Single-cell sequencing library preparation was performed using the 10x Genomics Chromium Controller and Chromium Next GEM Single Cell 3’ Kit v3.1 Chemistry Library, Gel Bead, CellPlex Multiplex, and Chip Kits (10x Genomics) according to the manufacturer’s protocols & instructions. A total of 5,000 to 20,000 cells were targeted per library and processed in parallel with replicates. Libraries produced were checked for quality control using the Agilent Tapestation, quantified using the KAPA library Quantification Kit, diluted as appropriate, then sequenced using an Illumina NextSeq 500 with paired-end sequencing and either single or dual indexing as appropriate for the library preparation. Cycling conditions for single index libraries were 28, 8, and 91 cycles for Read 1, i7 index and Read 2 respectively. Dual Index libraries were sequenced using cycling conditions 28, 10, 10, and 90 for Read 1, i7 index, i5 index and Read 2 respectively. The used resources are outlined in **Supplementary Note 2**.

### 4.4 Processing of scRNA-seq data

The *nextflow* (32) *nf-core* v2.11.1 pipeline scrnaseq with alignment option cellranger was employed for processing raw cynomolgus monkey and human sequencing reads. Here, the genome reference files *Macaca Fascicularis* 6.0 from Ensembl release 105 and *Homo Sapiens* GRCh38 from Ensembl release 108 were used. Ambient mRNA background correction was performed per sample and per species using R tool *SoupX* v1.6.2 (33) with normalized, log-transformed input and unique gene names.

### 4.5 Quality control

The python tool *DoubletDetection* v4.2 (34), the R tool *scDblFinder* v1.17.2 (35) and the R port of *Scrublet* (r_scrublet v0.1) (36) were used for identification of doublets. A cell was considered a doublet if at least two out of three doublet identifiers matched. Low-quality cells were identified via automatically determined thresholds based on the median absolute deviation (MAD) (37) for the log number of genes by counts and for the log total counts per sample and species. Cells with a mitochondrial count fraction exceeding 0.15% (cynomolgus monkey) or 15% (human) were considered low-quality cells. Further, we applied unsupervised clustering using *scanpy* v1 (38). function sc.tl.louvain v0.8.2 with resolution = 7 jointly on all samples per species. The relatively high resolution was chosen to produce more, smaller clusters, which supports the isolation of low-quality clusters and helps avoid excluding biologically relevant populations. We considered louvain clusters with a doublet fraction >0.25 as doublet clusters. Here, cells identified by at least one doublet detection tool were considered doublets. Louvain clusters with a low-quality cell fraction >0.25 were considered low-quality clusters. Eventually, we removed low-quality cells, low-quality clusters, doublets and doublet clusters. Low-quality clusters and doublet clusters were removed based on the rationale that cells within a neighborhood of many cells with quality issues might be compromised themselves, even if they do not exceed a quality threshold individually.

### 4.6 Cell type annotation

An initial cell type annotation at the single-cell level was obtained by applying the R-package based cell type annotation tool *Azimuth* v0.5.0 (20) within *Seurat* (39) v5.0.3 using reference dataset “pbmcref”. Cynomolgus monkey gene names were humanized based on their respective human orthologs (**Supplementary Table S1**). *Azimuth* provides hierarchical cell type predictions at several resolution levels, including broad level 1 categories and more granular level 2 subtypes. To optimize the balance between annotation granularity and statistical power for downstream analyses, we created an intermediate level 1.5 annotation by selectively using level 2 annotations for specific cell types while retaining level 1 annotations for others. From level 2, we retained: “Platelet”, “NK Proliferating”, “CD4 Proliferating”, “CD8 Proliferating”, “CD14 Mono”, “CD16 Mono”, “Plasmablast”, “gdT”, “MAIT”, and “dnT”. All remaining cell types used their corresponding level 1 annotations. For refinement and obtaining a more robust annotation on cluster level, we utilized *scVI* (19) implemented in *SeuratWrappers_0.3.5* to jointly embed cynomolgus monkey and human data after comparing standard integration algorithms implemented in SeuratWrappers_0.3.5 and Seurat v5.0.3. We performed clustering using *Seurat* function FindClusters with resolution = 4 on the *scVI* embedding and assigned each resulting cluster to the majority *Azimuth* cell type label within that cluster. A lower clustering resolution was used compared to the QC step to obtain more homogeneously annotated regions and avoid over-fragmentation. The expression levels of selected marker genes for each cell type were visualized to validate the annotation at the species level.

### 4.7 Cell type proportions

We analyzed cell type proportions in cynomolgus monkeys and humans at baseline (0h) and at timepoints 6h and 24h following *in vitro* anti-*CD3*/anti-*CD28* T Cell activation. Credible compositional changes in cell type composition were identified using *scCODA* (40) v0.1.9 in python. Automatic reference cell type selection was implemented by setting automatic reference_cell_type=“automatic” within the *scCODA* CompositionalAnalysis function, which resulted in B cells being selected as the reference in cynomolgus monkeys and CD8^+^ T cells in humans. Further, a false discovery rate (FDR) ≤ 25% threshold was applied to control for multiple testing within species.

### 4.8 Cross-species modelling of transcriptomic data using variational autoencoders

We used *scGen* v2.0.0 (18) variational autoencoder (VAE) implementation to jointly model cynomolgus monkey and human gene expression data, considering the top 2,000 highly variable genes per species with normalized and log1p-transformed (log(1 + x)) counts as input. We followed a systematic approach using ortholog information (**Supplementary Table S2**) from Ensembl release 111 to define a unified gene nomenclature. When a cynomolgus monkey gene had multiple human orthologs, we duplicated the respective entries to ensure that each human ortholog is matched with the corresponding cynomolgus monkey gene. Similarly, when a human gene had several cynomolgus monkey orthologs, we replicated the human gene entry to align it with all relevant cynomolgus monkey orthologs. If a gene was present in both species but not found in the ortholog table, we retained the human gene name. This approach aimed to preserve annotated ortholog relationships between cynomolgus monkeys and humans as comprehensively as possible when methodologically feasible. For analyses requiring 1:1 mappings (i.e. when counting unique genes, e.g. in differential gene expression analysis and cell-cell communication analysis), we retained the highest expressed ortholog when multiple orthologs were present. While preserving multiple orthologs may introduce some redundancy in the input space, it helps avoid discarding relevant biological signals. Moreover, the compact, low-dimensional representations learned by VAEs focus on dominant variation, implicitly reducing the impact of input redundancy.

#### 4.8.1 VAE model architecture

We mainly followed the recommended architecture of the *scGen* model, where both encoder and decoder consist of two hidden layers (800 nodes each) that are followed by batch normalization, leaky ReLU activation (negative slope 0.01) and dropout (p=2). As input to the VAE served the unified gene nomenclature based on the highly variable genes, as described above. Through hyperparameter tuning, we identified suitable parameters for the latent dimensionality and batch size for training. Using a random subset (1,000 cells) drawn from the full dataset, which spans all timepoints and cell types from both cynomolgus monkey and human data, we split the data into a training set (800 cells) and a test set (200 cells). We then trained VAE models across combinations of latent dimensions and batch sizes. Next, we evaluated these models on unseen data from the test set and determined the best combination - 10 latent dimensions and a batch size of 8 - based on the lowest reconstruction error calculated using mean squared error (**Figure 1C**, **Supplementary Table S3**). Training was conducted for up to 100 epochs, incorporating early stopping with a patience of 25 epochs to prevent overfitting. When training *scGen* VAE models, we observed that the training and validation loss curves decreased and converged at similar levels in all cases, indicating that the chosen architecture effectively learned and avoided overfitting (**Supplementary Figure S1**). This architecture was then used for training cell type-specific VAE models, for which we required a minimum of 1,000 cells per cell type to be included in the analysis. The cell type-specific VAE models were jointly trained on all available timepoints within each cell type.

### 4.9 Species- and temporal shifts in VAE latent space

We computed the species-shift vector δ**_species_** in the latent space of trained, cell type-specific VAE models by determining the difference vector between the mean latent representations of human and cynomolgus monkey cells at baseline (0h). This vector δ**_species_** approximates the transition from cynomolgus monkey to human in VAE latent space independent of *in vitro* anti-*CD3*/anti-*CD28* T Cell activation. Similarly, within each species and for each cell type, we calculated temporal-shift vectors δ**_temporal_** by forming pairwise the difference vectors between the mean latent representations at baseline 0h and subsequent timepoints 6h and 24h. The temporal-shift vectors δ**_temporal_**approximate the transcriptomic changes over time in VAE latent space at the cell type level within each species.

### 4.10 Application of VAE models, species- and temporal shifts to unseen data

First, we examined whether cell type-specific species-shift-vectors δ**_species_** can be applied to unseen data, thereby assessing their generalizability. For this, we split the data into a training set which included the replicates *cyno1* (11,465 cells) and *human1* (9,962 cells), and a test set, which included the replicates *cyno2* (11,892 cells) and *human2* (11,750 cells). Here, the training of the cell type-specific VAE models was conducted on the training set. The model architecture and training parameters were set to be identical to those described in section VAE model architecture. Per cell type, we calculated the species-shift-vector δ**_species_** as described in section Species- and temporal shifts in VAE latent space. Next, we embedded the cynomolgus monkey controls (0h) from the test set and applied the species-shift-vector δ**_species_**from the training set to obtain an *in-silico* humanized embedding. Subsequently, we used the VAE decoder to map the *in-silico* humanized embedding back to gene expression space. We quantified the similarity in gene expression between the *in-silico* humanized cynomolgus monkey controls (0h), the observed cynomolgus monkey controls (0h) and the observed human controls (0h) from the test set using spearman rank correlation of mean gene expression ranks. We applied the same procedure with a training-test split according to replicates as well as a random training-test split with a test size of 50% to evaluate the generalizability of the temporal-shift vectors δ**_temporal_** in VAE latent space within cynomolgus monkeys and humans to unseen data.

### 4.11 Comparison of *scGen* and *LDVAE* for *in silico* humanization

We compared the generalizability to unseen data of the cell type-specific species-shift-vectors δ**_species_** derived from the latent representation of trained *scGen* VAE models to those derived from the *scvi-tools* (19) v0.14.6 *linearly-decoded variational autoencoder (LDVAE)* models (21). The *LDVAE* model includes a linear decoder which establishes a direct and interpretable link between the latent representation and gene expression space. We mainly followed the recommended *LDVAE* architecture (**Supplementary Figure S2A**), determining suitable latent dimensionality and batch size for training through hyperparameter tuning as for the *scGen* model (**Supplementary Table S4**).

### 4.12 Feature importance in trained *scGen* VAE models

We performed a *post-hoc* analysis to assess the feature importance of individual genes within trained *scGen* VAE models (16). The premise was that genes that induce a substantial change in the latent embedding upon slight perturbation are integral to VAE latent representation. In our heuristic approach, we perturbed one gene at a time by shifting the gene input value towards the species-specific mean, adjusting the magnitude of the shift according to the species-specific variance of the respective gene. The input values of all other genes remained unchanged. We then measured the feature importance by calculating the mean Euclidean distance between perturbed and unperturbed embeddings across the latent dimensions.

### 4.13 Differential gene expression and pathway enrichment

Differential gene expression (DGE) analysis was conducted within each species at the cell type level using *edgeR* (41) v3.40.2. We compared gene expression at timepoints 6h and 24h following *in vitro* T cell activation to gene expression at baseline (0h). The analysis was restricted to cell types that had a minimum of five cells per timepoint in all replicates. In a standard workflow, we constructed a DGEList object and applied the R function filterByExpr with min.prop=0.7 parameter to select genes with sufficient counts for inclusion in the analysis. Library Size Normalization was performed with the R function calcNormFactors. To control for multiple testing, the false discovery rate (FDR) according to Benjamini and Hochberg was applied. We used *FDRtool* (22) v1.2.18 in R to quantify the fraction of the transcriptome differentially regulated at timepoints 6h and 24h following T cell activation compared to baseline (0h) at the cell type level. To compare differential gene expression between cynomolgus monkeys and humans, we assessed the intersection of significantly regulated genes at FDR ≤ 20% in both species for each DGE condition. We chose this cutoff to optimize the sensitivity since different effect size levels can be expected across species. Additionally, we analyzed the fraction of these genes regulated in the same direction (i.e. up- or downregulation). The statistical significance of this gene regulation in the same direction between species was assessed using the R function prop.test with a two-sided alternative hypothesis and 95% confidence level.

Pathway enrichment analysis was conducted at the cell type level for timepoints 6h and 24h following *in vitro* T cell activation against baseline (0h). For this, the *GSEApy* (42) *Enrichr* (43) API was used in python, assessing databases Reactome, WikiPathway, GO and KEGG. We filtered the genes from DGE with an FDR ≤ 20% and selected per species and condition the top 200 genes based on absolute log fold change as foreground. To focus on the most relevant pathways, we applied an FDR ≤ 5% filter and required pathways to contain at least 2 foreground genes. To reduce redundancies, only the pathway with highest pathway enrichment (odds ratio) was retained in cases where multiple pathways had identical foreground gene sets. For visualizations, we considered the log odds ratio between expected and observed enrichment as pathway enrichment factor. The top enriched pathways in VAE latent representations were identified in the same manner but using the top 200 genes from VAE feature importance analysis as foreground instead of the top 200 genes from DGE. When comparing the top enriched pathways in VAE models with those enriched in DGE, we used log odds ratio as pathway enrichment factor and did not apply any filtering to the pathways derived from DGE. Further, we investigated the overlap of pathways enriched in both species in DGE to determine if identical foreground genes were responsible for the observed co-enrichment. Similarly, we assessed whether co-enriched pathways in VAE feature importance analysis and DGE shared the same foreground genes. To quantify the extent of similarity of this overlap, we calculated Jaccard index, measuring the proportion of intersection over union for the sets of foreground genes in co-enriched pathways.

### 4.14 Cell type-specific cluster analysis

For CD8^+^ T cells and NK cells, we analyzed changes in the abundance of scRNA-clusters derived from the *scVI* embedding (see section Cell type annotation). The proportion of each subcluster across time was analyzed using *scCODA* as outlined in section Cell type proportions. For NK cells in cynomolgus monkeys, reference cluster 23 was manually selected while automatic reference selection was applied in all other cases. For specific subclusters, we calculated differentially expressed genes against the remaining cells within each species for the corresponding cell type. We used Seurat’s FindMarkers() function with method = ‘wilcox’ to identify cluster-specific genes within each cell type. Genes were ranked by average log2-Fold-change and gene set enrichment was done using ReactomePA 1.48.0 with function gsePathway().

### 4.15 Cell-cell communication

To identify cell signaling patterns before and after immune activation, we performed cell-cell communication (CCC) analysis. For this, we considered all cells passing quality control (QC) as input without applying an initial gene filter. Cynomolgus monkey gene names were mapped to their respective human orthologs (**Supplementary Table S10**). In cases with multiple human orthologs, the respective rows were duplicated and expression values for entries with the same human gene name were summed. CCC was analyzed at baseline (0h) and at timepoints 06h and 24h following T cell activation at the cell type- and species-level using LIANA (23) v0.1.9 in a python v3.12.3 environment. The raw read matrix was normalized using scanpy (38) v1.10.1 and LIANA was run using all methods for ligand-receptor-interaction prediction provided by the LIANA+ package with parameter return_all_lrs=True and default parameters otherwise. We analyzed both the quantitative patterns of signaling activity and the conservation of specific ligand-receptor pairs across species. To assess cross-species conservation of CCC patterns, we calculated the percentage of ligand-receptor pairs that were significantly active in both species versus those specific to individual species at a magnitude rank and specificity rank ≤ 0.01. Network visualization was performed using the igraph 2.0.3 package in R, where nodes represent cell types and directed edges represent ligand-receptor interactions, filtering magnitude rank and specificity rank ≤ 0.001 to visualize the most stringent relations. Edge weights were scaled according to the sum of log10-transformed magnitude ranks. To test whether ligand-receptor pairs overlapped more than expected by chance between species, we applied Fisher’s exact test using cell type-specific all reported ligand-receptor pairs in LIANA regardless of significance as background.

An overview of all analysis steps is provided in **Figure 1B**.

## Data Availability

All scripts to reproduce figures and result tables are available at GitHub: https://github.com/GenStatLeipzig/Deep-learning-aided-inter-species-comparison-inIn review cynomolgus-monkey-and-humans.git. Data for this study are deposited in Zenodo under link: https://zenodo.org/records/17078934?preview=1&token=eyJhbGciOiJIUzUxMiJ9.eyJpZCI6IjVkNWUxMWM3LTAxMzQtNDlkYS1hOTkzLWU3MWMxZmMwYzM4YyIsImRhdGEiOnt9LCJyYW5kb20iOiJiNWMyYTcxYzY2ZDAxNjYxOGEzMTQ1NDI1YzE5YjdmNyJ9.3gtYgogVWt4ZfQhFMlqhLkdPnHwjTfT9ify3j1GSvb4vMHoQu3vM5L7oHyq9xc0zm6YjkpgAIAfMZfwddBpiw.
