# Supplementary material for: Deep learning-aided inter-species-comparison reveals shared and distinct molecular patterns in cynomolgus monkey and humans following non-specific T cell activation
Source: Front Immunol. 2025 Dec 12;16:1603716. doi: 10.3389/fimmu.2025.1603716 (PMC12741143; doi:10.3389/fimmu.2025.1603716)
Supplement: Supplementary file 1 [file DataSheet1.pdf]

Deep learning-aided inter-species-comparison reveals shared and distinct molecular patterns in cynomolgus monkey and humans following non-specific T cell activation

Vincent D Friedrich[1,2], Kari Neier[3], Kristina Müller[1,2], Birgit Fogal[3], Zuzana Loncova[4], Michael Rade[5], Muhammad Shoaib[6], Ulrike Köhl[7,5,2,9], Kathleen Hoyt[3], Parimal Pande[3], Lily Blanchard[3], Ernest Raymond[3], Markus Scholz[1,8], Kristin Reiche[5,9,2,+,*], Holger Kirsten[1,2+,*]

[1]University of Leipzig, Institute for Medical Informatics, Statistics, and Epidemiology, Leipzig, Germany.
[2]Center for Scalable Data Analytics and Artificial Intelligence (ScaDS.AI), Leipzig, Germany.
[3]Boehringer Ingelheim Pharmaceuticals, Inc., Ridgefield CT 06877, USA.
[4]Biocenter, Institute of Bioinformatics, Medical University of Innsbruck, 6020 Innsbruck, Austria.
[5]Fraunhofer Institute for Cell Therapy and Immunology, Leipzig, Germany.
[6]Luxembourg Centre for Systems Biomedicine, University of Luxembourg, 6 Avenue du Swing, L-4367 Belvaux, Luxembourg.
[7]Cancer Center Central Germany (CCCG) Leipzig-Jena, University Hospital of Leipzig, Leipzig, Germany.
[8]University of Leipzig, Faculty of Mathematics and Computer Science, Leipzig, Germany.
[9]Institute for Clinical Immunology, Leipzig University, Johannisallee 30, 04103, Leipzig, Germany.

[+]co-last authors

*Corresponding Authors:
Holger Kirsten, Dr. rer. nat.
Institute for Medical Informatics, Statistics, and Epidemiology (IMISE)
Haertelstraße 16-18, 04107 Leipzig, Germany
E-mail: holger.kirsten@imise.uni-leipzig.de / Phone: +49 341 9716100

Kristin Reiche, Dr. rer. nat.
Department of Medical Bioinformatics, Fraunhofer Institute for Cell Therapy and Immunology,
Perlickstraße 1, 04103 Leipzig, Germany, Leipzig, Germany
E-mail: kristin.reiche@izi.fraunhofer.de / Phone: +49 341 35536-5223

Supplementary Material

1 Supplementary Data

1.1 Supplementary Note 1

Reagents used for PBMC stimulation

The following reagents were used for the stimulation of cynomolgus monkey PBMCs using anti-*CD3*/anti-*CD28* antibody:

- SepMate-50 Tubes (Stemcell Tech Cat# 85450)

- Lymphoprep Solution (Stemcell Tech Cat# 07851)
- 1xPBS + 2% FBS (Stemcell Tech Cat# 07905)
- 1xPBS (Thermofisher Sci Cat# 10010023)
- RPMI 1640 with L-Glutamine (Thermofisher Sci Cat# 61870036)
- Streptomycin/Penicillin (Thermofisher Sci Cat# 10378016)
- Anti-CD3 Antibody SP34 (BD Biosciences Cat# 551916)
- Anti-CD28 Antibody (eBiosciences Cat# 16-0289-85)
- Microcentrifuge Tubes
- 15ml, 50ml Conical Tubes
- 12-Well Tissue Culture Plates

The following reagents were used for the stimulation of human PBMCs using anti-*CD3*/anti-*CD28* antibody:

- SepMate-50 Tubes (Stemcell Tech Cat# 85450)
- Lymphoprep Solution (Stemcell Tech Cat# 07851)
- 1XPBS + 2% FBS (Stemcell Tech Cat# 07905)
- 1xPBS (Thermofisher Sci Cat# 10010023)
- RPMI 1640 with L-Glutamine (Thermofisher Sci Cat# 61870036)
- Streptomycin/Penicillin (Thermofisher Sci Cat# 10378016)
- Heat Inactivated Fetal Bovine Serum (Thermofisher Sci Cat# A3840101)
- Anti-CD3 Antibody SP34 (BD Biosciences Cat# 551916)
- Anti-CD28 Antibody (eBiosciences Cat# 16-0289-85)
- Microcentrifuge Tubes
- 15ml, 50ml Conical Tubes
- 96-Well Tissue Culture Plates

1.2 Supplementary Note 2

Key resources used for single-cell sequencing

REAGENT or RESOURCE	SOURCE	IDENTIFIER
Critical commercial assays		
Next GEM Single Cell 3' GEM Kit v3.1	10x Genomics	1000123

Next GEM Single Cell 3' Gel Bead Kit v3.1	10x Genomics	1000122
Single Cell 3' v3.1 Gel Beads	10x Genomics	2000164
Chromium Next GEM Chip G	10x Genomics	2000177
3' CellPlex Kit Set A	10x Genomics	1000261
Dual Index Plate TT Set A	10x Genomics	3000431
Dual Index Plate NN Set A	10x Genomics	3000482
Chromium i7 Index Plate	10x Genomics	120262, 220103
Library Construction Kit	10x Genomics	1000190
KAPA Library Quant Kit	Illumina	KK4873
NextSeq 500/550 Hi Output kit v2.5 (150 Cycles)	Illumina	15057931, 15057941, 15058251, 15009740, 20022408, 15041963, 15017397, 15026770
SPRIselect Reagent	Beckman Coulter	B23318
High Sensitivity D5000 Tapestation Cassette	Agilent Technologies	5067-5592
High Sensitivity D5000 Tapestation Buffer	Agilent Technologies	5190-7745
High Sensitivity D5000 Tapestation Ladder	Agilent Technologies	5190-7747
High Sensitivity D1000 Tapestation Cassette	Agilent Technologies	5067-5584
High Sensitivity D1000 Tapestation Buffer	Agilent Technologies	5067-5603
High Sensitivity D1000 Tapestation Ladder	Agilent Technologies	5067-5587

Chemicals, peptides, and recombinant proteins		
DNase I	Roche/Sigma	10104159001
Tris-HCl (pH 7.4)	Sigma Aldrich	T2194
10% BSA in DPBS	Sigma Aldrich	A1595-50ML
Low TE Buffer	Thermo Fisher	12090-015
SPRIselect Reagent	Beckman	B23318
High Sensitivity D5000 Tapestation Cassette	Agilent	5067-5592
High Sensitivity D5000 Tapestation Buffer	Agilent	5190-7745
High Sensitivity D5000 Tapestation Ladder	Agilent	5190-7747
KAPA SYBR FAST qPCR Master Mix	Illumina	KK4600
10% Tween 20	Bio-Rad	1662404
Buffer EB	Qiagen	1014608

2 Supplementary Figures

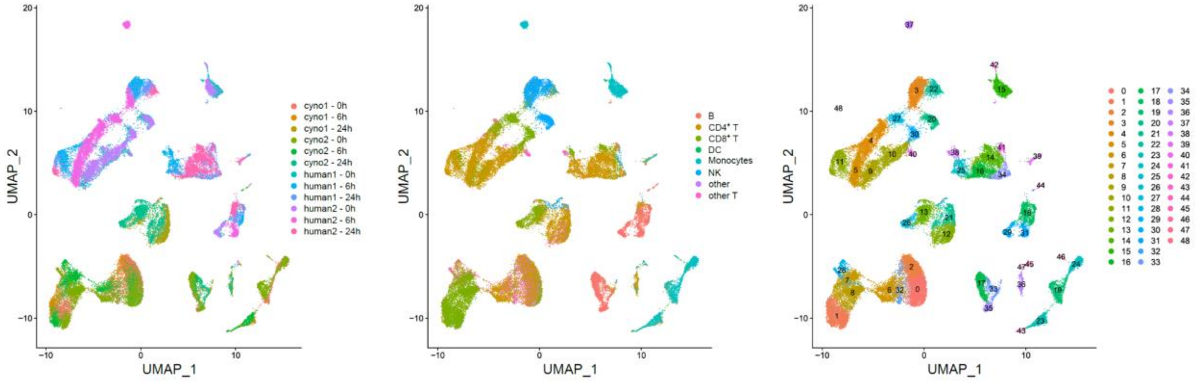

Supplementary Figure 1. Unintegrated data. Uniform manifold approximation and projection (UMAP) plot of unintegrated cross-species scRNA-seq data colored by species, replicate and timepoint post anti-*CD3*/anti-*CD28* T Cell activation (left panel), by cell type based on Azimuth using automated annotation of individual cells applying reference *pbmcref* (middle panel) and by clustering (right panel). cyno: cynomolgus monkey. h: hours.

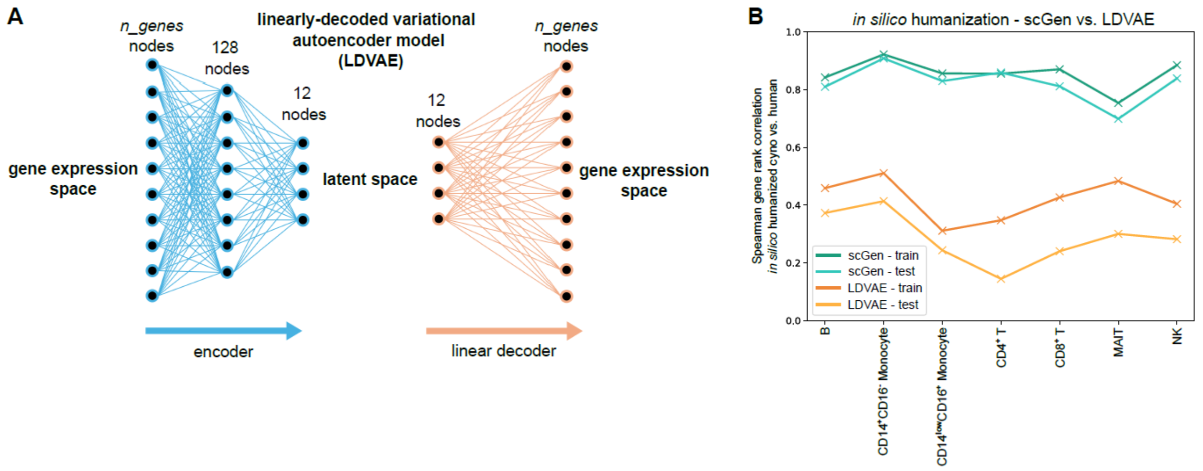

Supplementary Figure 2. Comparative analysis of *scGen* and *linearly-decoded variational autoencoder model (LDVAE)* models. (**A**) Schematic of the used *LDVAE* model architecture. (**B**) Performance evaluation of *scGen* and *LDVAE* model for *in-silico* humanizing cynomolgus monkey controls (0h) at the cell type level, assessed with Spearman gene rank correlation. Both training and test set contain one replicate per species.

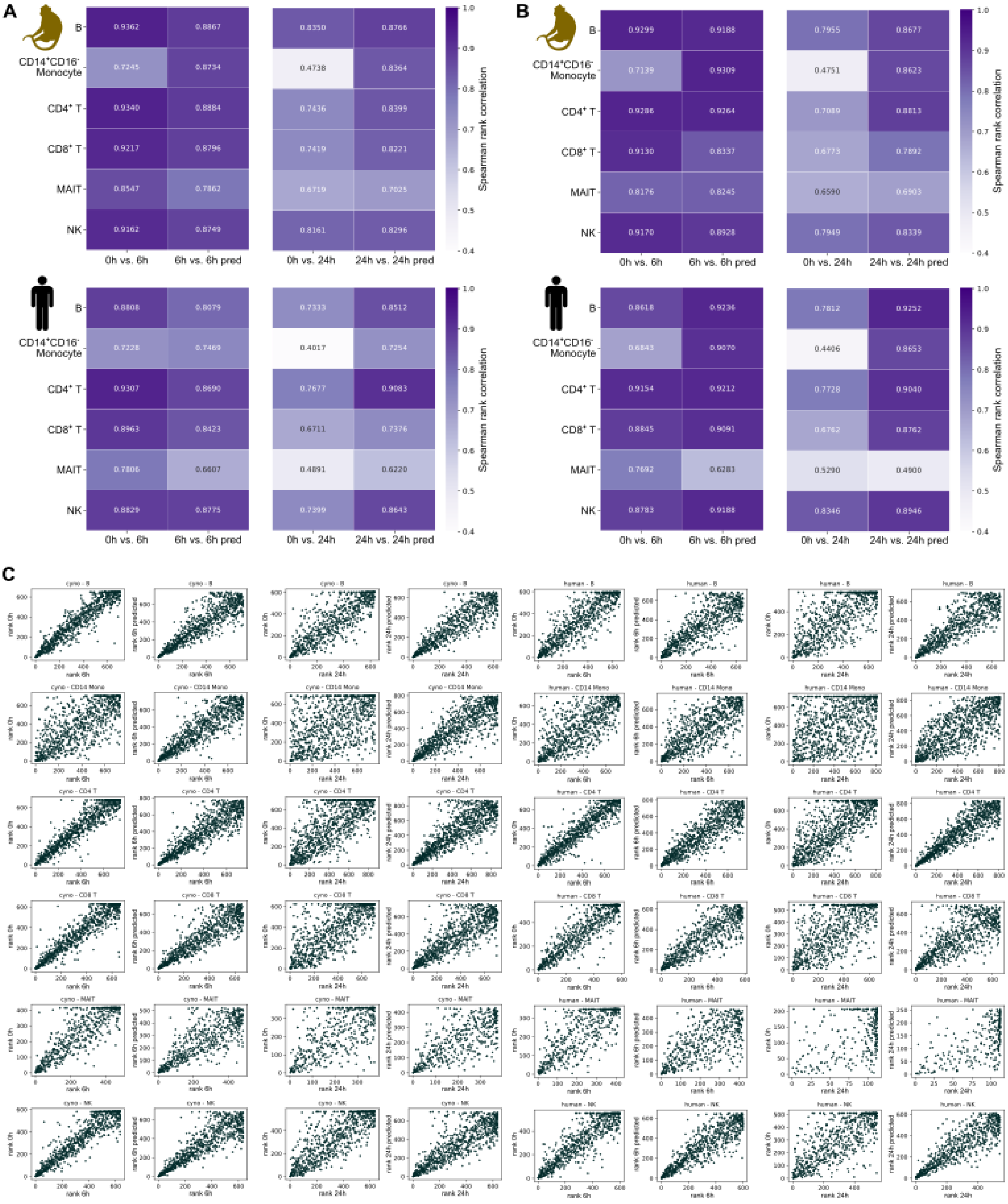

Supplementary Figure 3. Generalization of temporal shift vectors in VAE latent space to unseen data. This figure illustrates the predictive application of temporal shift vectors, derived from baseline (0h) to timepoints 6h and 24h using two different training/test splits: (**A**) training and test set consist each of one replicate per species, and (**B**) training and test set are created through a random 50:50 cell split. Spearman's gene rank correlation assesses the generalizability of the latent shift vectors by comparing the predicted states at 6h and 24h to both the observed baseline (0h) and the corresponding observed timepoints. Results are shown separately for cynomolgus monkey (top panels) and human (bottom panels)**.** In most cases, generalizability of the latent shift vectors was higher in the random 50:50 cell split

scenario, as indicated by the darker colors in the right column of panel (**B**) compared to the right panel of (**A**). (**C**) Scatter plots comparing gene expression ranks on the test set when split according to replicates. h: hours. pred: predicted.

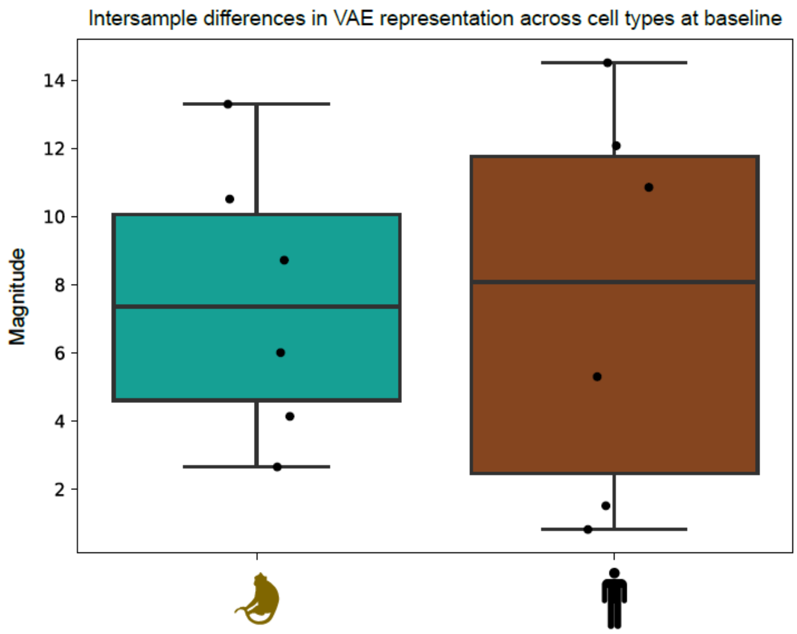

Supplementary Figure 4. Intersample differences within species in VAE representation across cell types at baseline. Boxplot showing the magnitude (L2 norm) of shift vectors across cell types between mean latent representations of the two replicates within the same species in the VAE latent space of trained, cell type-specific *scGen* VAE models. As both cynomolgus monkeys are male, while the human samples include one male and one female, the comparable levels of variation suggest that sex is not a primary driver of baseline differences. Even though variation appeared to be slightly increased in humans, this was not of statistical significance (Levene test p-value > 0.1).

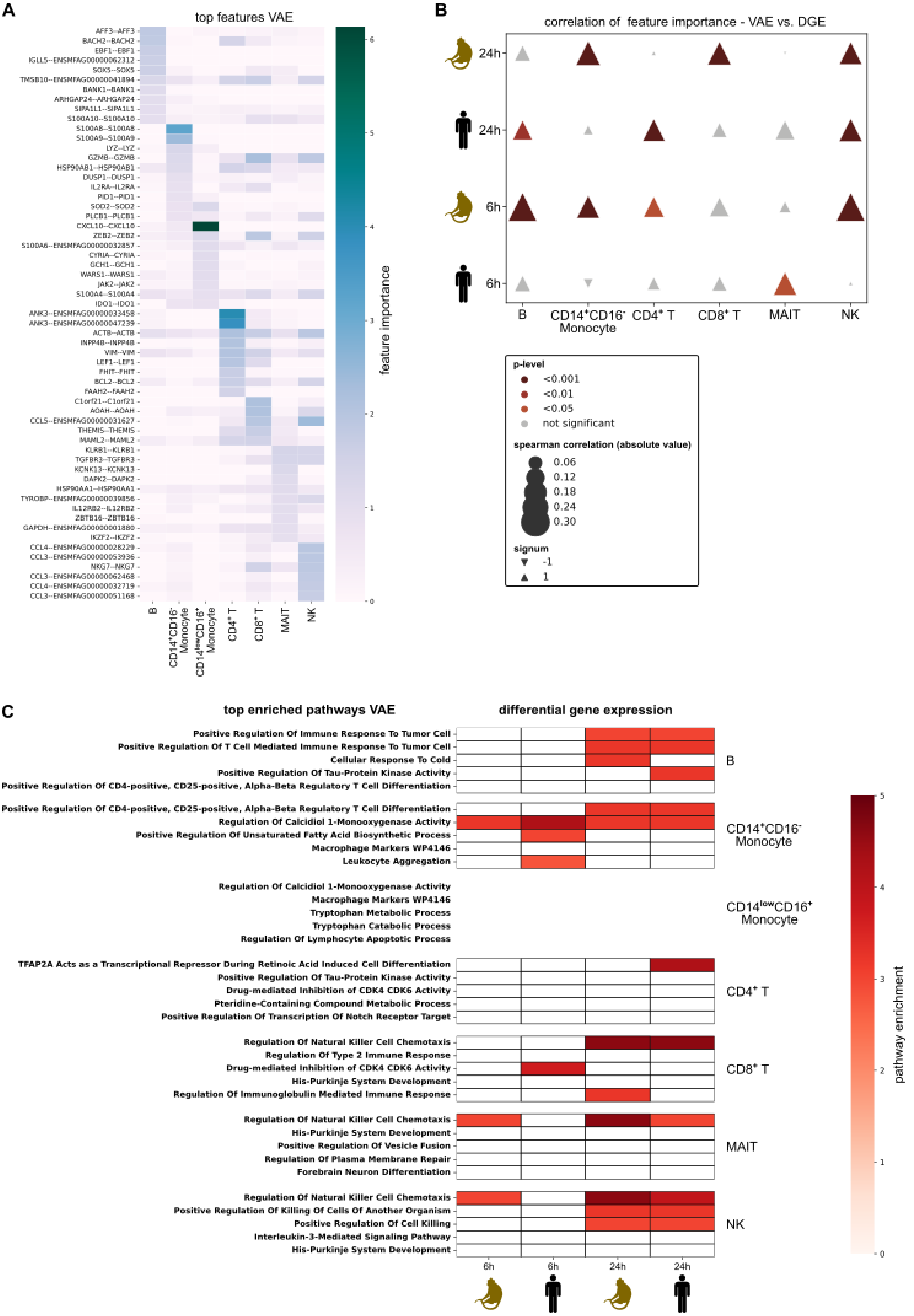

Supplementary Figure 5. Feature importance VAE. (**A**) Identification of top genes in cell-type specific VAE models via feature importance analysis. (**B**) Correlation between feature

importance in VAE and differential gene expression (DGE) for genes with FDR ≤ 50%, assessed using Spearman rank correlation. Genes were ranked by perturbation effect in the VAE and by p-value in the DGE analysis before correlation assessment. The color of the triangles represents the statistical significance (p-value) of the correlation, the size indicates the absolute value of the Spearman correlation, and the orientation shows directionality - triangles pointing upward represent positive correlation and triangles pointing downward negative correlation. (**C**) Top enriched pathways from VAE feature importance analysis compared with pathway enrichment factors derived from differential gene expression analysis, presented per species and timepoint. Pathway enrichment analysis was performed using the GSEApy Enrichr API with the databases Reactome, WikiPathway, GO, and KEGG. Pathway enrichment score, calculated as the log odds ratio, quantifies the deviation between observed and expected pathway enrichment. In cases of pathway co-enrichment between VAE feature importance analysis and DGE, at least one foreground gene was shared in all co-enriched pathways. The median Jaccard index, used to quantify the overlap of foreground genes per species and timepoint across cell types with VAE feature importance analysis, was lower for the 6h timepoints (0.375 for cynomolgus monkeys and 0.3875 for humans) compared to the 24h timepoints (0.444 for both species). A Jaccard index of 1 indicates complete overlap of foreground genes, while 0 indicates no overlap. For example, genes *CCL4*, *CCL3*, and *XCL1* were shared in the foreground of the *Regulation Of Natural Killer Cell Chemotaxis* pathway in both VAE feature importance analysis and the human and cynomolgus monkey 24h DGE in CD8 T cells. In VAE feature importance, the foreground additionally included *CCL5*, which was not in the foreground for DGE. h: hours.

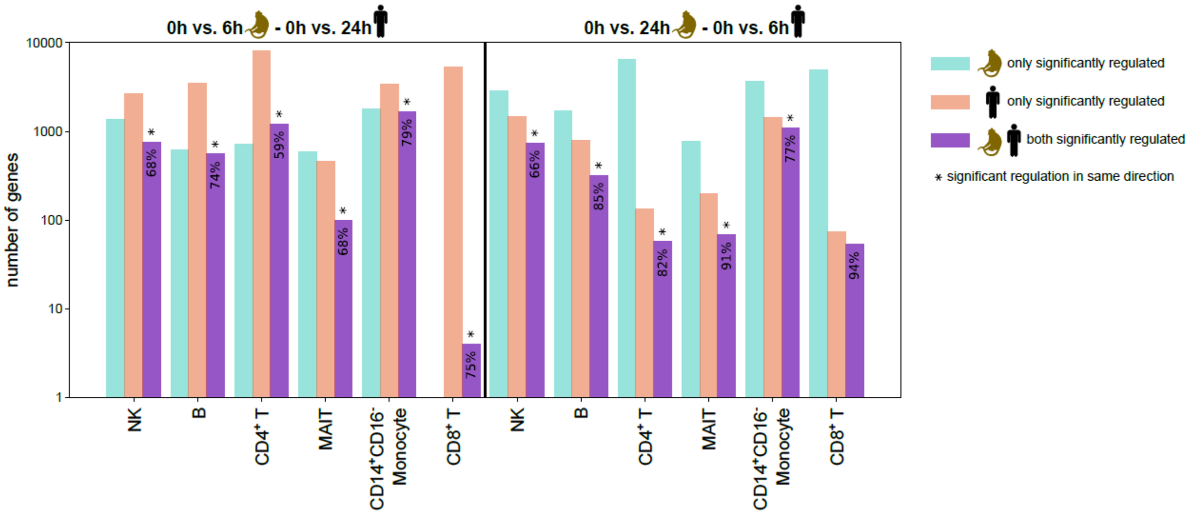

Supplementary Figure 6. Comparison of differentially expressed genes across species with asynchronous timepoints. Barplots showing the logarithmic number of differentially expressed genes (DGE) between species across timepoints. The left panel compares 0h vs. 6h in cynomolgus monkeys to 0h vs. 24h in humans, while the right panel compares 0h vs. 24h in cynomolgus monkeys to 0h vs. 6h in humans at FDR ≤ 20%. In the bar representing significant genes shared by both species, the percentage of genes regulated in the same direction is displayed. Statistical significance of this co-regulation ($p \leq 0.01$) is indicated by a star and was calculated using the prop.test function in R. h: hours.

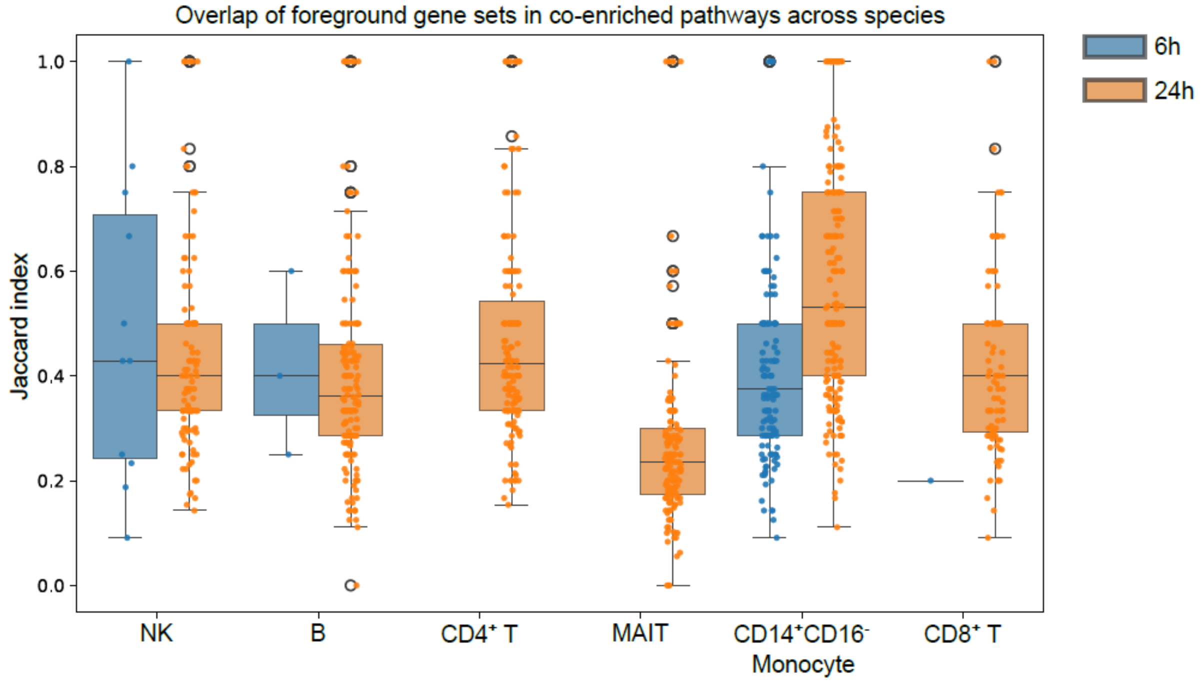

Supplementary Figure 7. Overlap of foreground gene sets in co-enriched pathways across species. Boxplot showing the Jaccard index for the overlap of foreground genes in co-enriched pathways at 6h (blue) and 24h (orange). Missing blue boxes indicate no overlapping enriched pathways across species in differential expression analysis. A clear time effect is present with typically more similar shared foreground genes 24h after activation. h: hours.

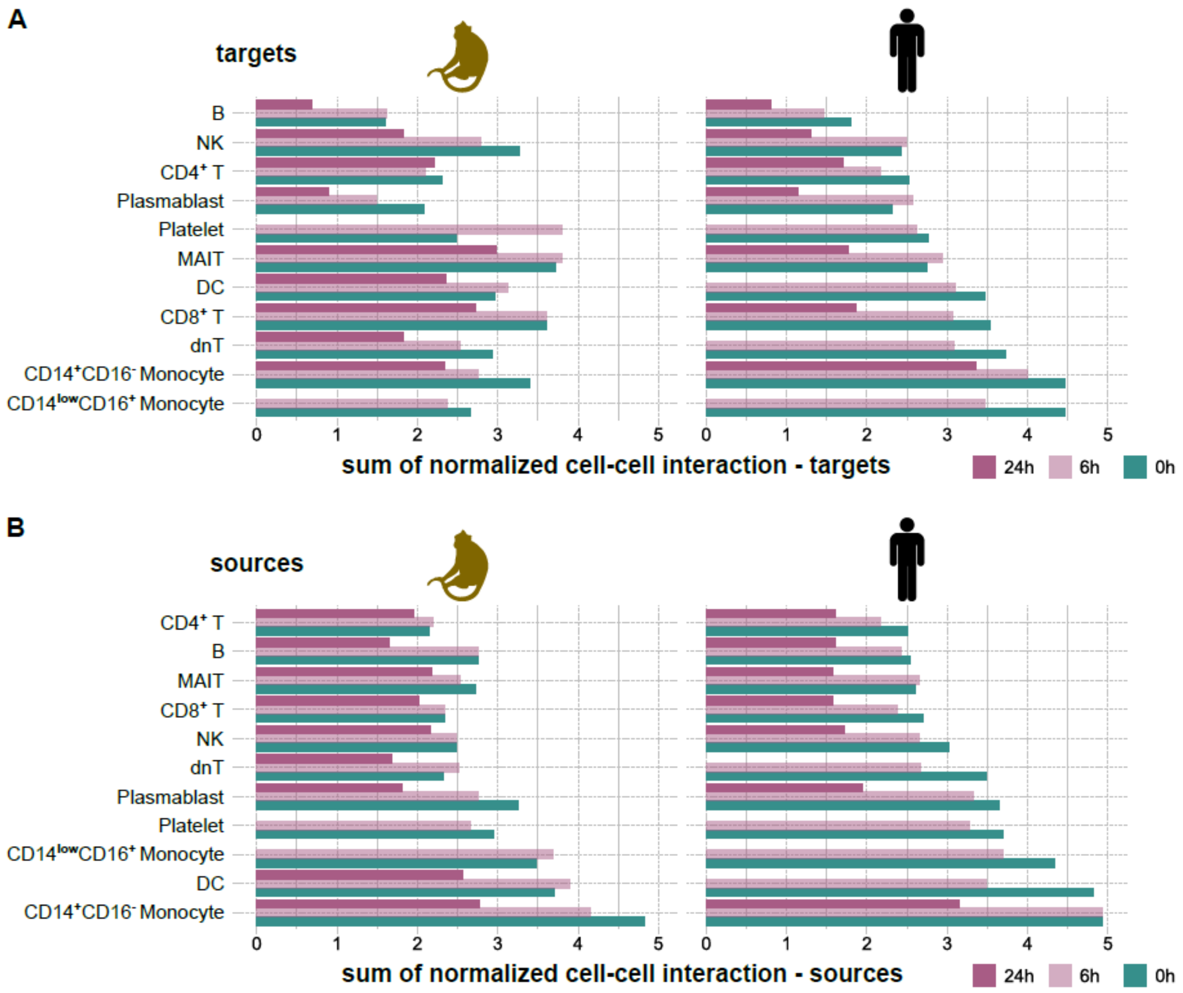

Supplementary Figure 8. Comparison of cell-cell signaling networks between human and cynomolgus monkey. (**A**) Target expression patterns across different immune cell populations shown as magnitude of cell-cell interactions at 0h, 6h, and 24h. (**B**) Corresponding sources expression patterns in the same cell populations. Cell-cell interaction strengths were quantified as sum of the normalized expression levels and normalized to the total number of receptors and Ligands included in the *LIANA* database per cell type for all source-target interactions with specificity rank p-value ≤ 0.05 and magnitude rank p-value ≤ 0.05. h: hours.

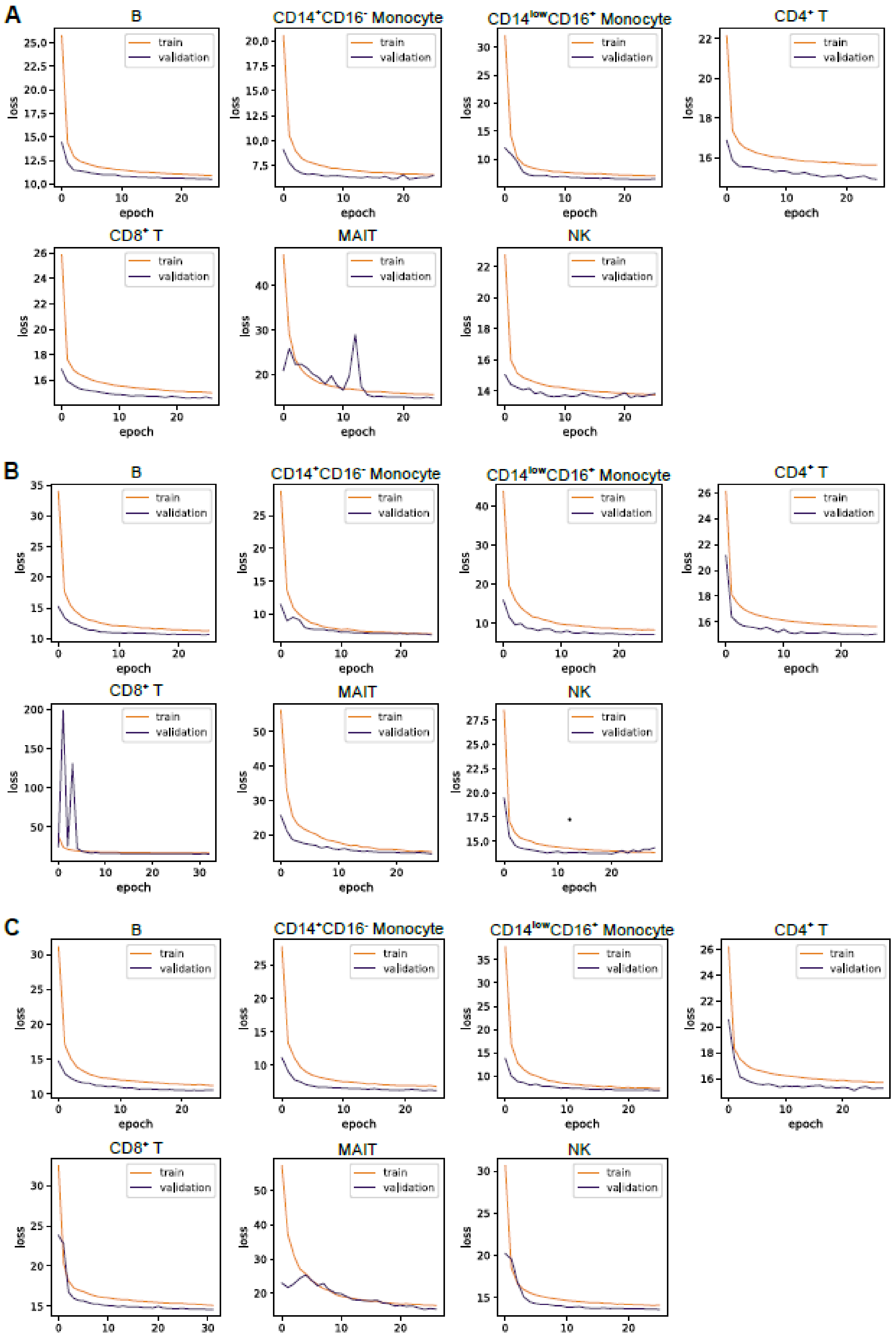

Supplementary Figure 9. Loss. Train and validation loss of cell type-specific *scGen* VAE models trained on different subsets of data: (**A**) full dataset, (**B**) one replicate of cynomolgus

monkey and human replicate each, (**C**) random 50% split of full dataset. Cell type-specific VAEs were trained for cell types with at least 1,000 cells.
